# Selective recognition of RNA G-quadruplex *in vitro* and in cells by L-aptamer–D-oligonucleotide conjugate

**DOI:** 10.1093/nar/gkae1034

**Published:** 2024-11-18

**Authors:** Haizhou Zhao, Hill Lam Lau, Kun Zhang, Chun Kit Kwok

**Affiliations:** Department of Chemistry and State Key Laboratory of Marine Pollution, City University of Hong Kong, 83 Tat Chee Avenue, Kowloon Tong, Hong Kong SAR, China; Department of Chemistry and State Key Laboratory of Marine Pollution, City University of Hong Kong, 83 Tat Chee Avenue, Kowloon Tong, Hong Kong SAR, China; Department of Chemistry and State Key Laboratory of Marine Pollution, City University of Hong Kong, 83 Tat Chee Avenue, Kowloon Tong, Hong Kong SAR, China; Department of Chemistry and State Key Laboratory of Marine Pollution, City University of Hong Kong, 83 Tat Chee Avenue, Kowloon Tong, Hong Kong SAR, China; Shenzhen Research Institute of City University of Hong Kong, 8 Yuexing 1st Road, Shenzhen Hi-Tech Industrial Park, Shenzhen, 518057, China

## Abstract

RNA Guanine-quadruplexes (rG4s) are important nucleic acid structures that govern vital biological processes. Although numerous tools have been developed to target rG4s, few specific tools are capable of discerning individual rG4 of interest. Herein, we design and synthesize the first L-aptamer–antisense oligonucleotide (ASO) conjugate, L-Apt.4–1c-ASO15nt_*(APP)*_, with a focus on recognizing the amyloid precursor protein *(APP)* rG4 region as an example. The L-aptamer module binds with the rG4 structure, whereas ASO hybridizes with flanking sequences. Together, these two modules enhance the precise recognition of *APP* rG4. We demonstrate that the L-Apt.4–1c-ASO15nt*_(APP)_* conjugate can interact with the *APP* rG4 region with sub-nanomolar binding affinity, and distinguish *APP* rG4 from other G4s and non-G4s *in vitro* and in cells. We also show that L-Apt.4–1c-ASO15nt*_(APP)_* can inhibit APP protein expression. Notably, we investigate the inhibitory mechanism of this newly developed tool, and reveal that it controls gene expression by hindering DHX36 protein from unraveling the rG4, as well as by promoting translational inhibition and RNase H-mediated mRNA knockdown activity. Our novel L-aptamer–ASO conjugate tool not only enables the specific recognition of rG4 region of interest, but also allows efficient gene control via targeting rG4-containing transcripts in cells.

## Graphical Abstract

**Graphical Abstract:**
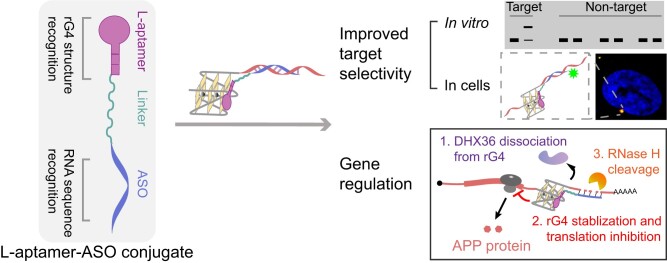

## Introduction

**Figure 1.**
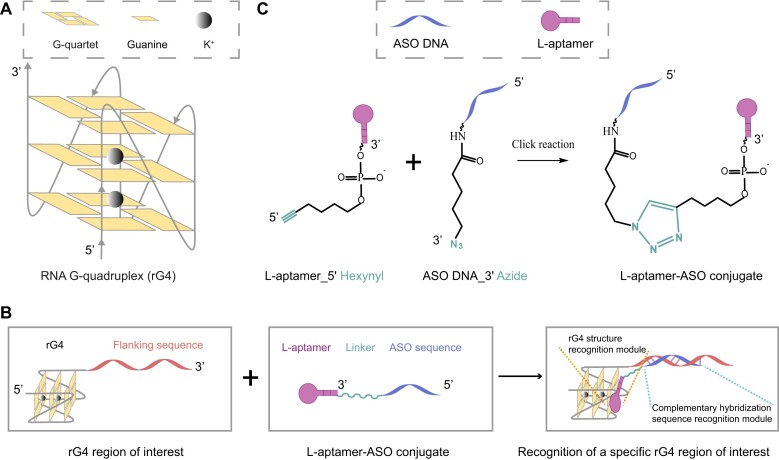
Rational design of L-aptamer–antisense oligonucleotide (ASO) conjugate for recognizing specific rG4 region of interest. (**A**) Schematic illustration of an rG4 structure. The G-quartets are further stabilized by K^+^. (**B**) The components of the rG4 region of interest and L-aptamer–ASO conjugate, and the principle for molecular recognition. The L-aptamer module is used to recognize the rG4 motif via structural recognition, and the ASO module is employed to recognize the flanking sequence near the rG4 motif via sequence recognition. By combining the L-aptamer module with the ASO module, an L-aptamer–ASO conjugate that utilizes both the structural recognition and sequence recognition mechanisms can be generated for better target binding affinity and selectivity. (**C**) The conjugation of the L-aptamer module and ASO module via chemical ligation. L-aptamer and ASO are modified with 5′ hexynyl and 3′ azide functional groups respectively, followed by Cu (I)-catalyzed azide–alkyne click reaction to produce the L-aptamer–ASO conjugate.

Guanine quadruplex (G4) structures are DNA and RNA secondary structural motifs with important chemical and biological roles (1,2). G4 structures are formed by guanine (G)-rich sequences, in which two or more stacks of G-quartets are stabilized by central metal cations, most commonly potassium ions (K^+^) or sodium ions (Na^+^) (1,3) (Figure 1A). The distinctive folding topology and chemical properties of G4s make them useful molecular scaffolds for structural investigations and tool development (4,5). Historically, DNA G4s (dG4s) have been more frequently studied than RNA G4s (rG4s) (1,6). Nonetheless, with the advance of technology and increasing interest in RNA structure and biology, the prevalence, formation, dynamics and function of rG4s have been increasingly reported in the past years (6). Recent studies have demonstrated that rG4s can be found in a range of species, including bacteria, the malaria parasite, African trypanosomes, *Leishmania tarentolae*, *Trypanosoma cruzi*, plants, humans and others (7–13). Moreover, rG4s have been identified in many RNA classes, such as messenger RNA (mRNA), microRNA and long non-coding RNA, among others (14–19). Other reports have suggested that rG4s play key roles in the regulation of translation (14,20–22), alternative polyadenylation (23), alternative splicing (24–26), RNA modification (27), RNA transport (28), mRNA stability (29), RNA editing (13,30) and RNA phase separation (31). Emerging research suggests that rG4s are linked to health and various diseases (21,32–35), such as cancers, neurodegenerative diseases and viral pathogenesis.

Among the reported rG4 sequences in humans, the amyloid precursor protein (*APP)* rG4 is one of the most studied due to the importance of the *APP* gene's association with Alzheimer’s disease (36,37). *APP* rG4 exists in the 3′ untranslated region (UTR) of *APP* mRNA, and negatively regulates *APP* gene expression (21,38). The direct regulation of *APP* expression can therefore likely be achieved by developing tools to target this rG4 secondary structure. The commonly used approaches for G4 targeting include the development of G4-specific small-molecule ligands (39), peptides (40) or antibodies (41). However, the binding specificity of these tools is typically only sufficient to distinguish between G4 structures and double/single-stranded DNA or RNA. Recently, our group developed a new type of G4 targeting tool, the L-RNA aptamer (Spiegelmer). L-RNA aptamers form by the folding of unnatural single-stranded mirror L-RNA nucleotides, and they cannot undergo Watson–Crick base pairing with the natural D-nucleotides. As such, L-RNA aptamers recognize their D-RNA target primarily by its 3D structure, giving them high specificity (42–45). Using our newly developed methods, rG4-systematic evolution of ligands by exponential enrichment (rG4-SELEX) and G4-SELEX-seq, we have developed several L-RNA aptamers with better G4 specificity than the above-mentioned tools (46–50). For example, L-Apt12-6 can selectively recognize parallel G4s over antiparallel/hybrid G4s and non-G4 structures (49). This has been applied to suppress gene expression in cells via targeting the c-*kit 1* dG4 structure (49). Another L-aptamer, L-Apt.4–1c, is a general rG4 binder, and can efficiently distinguish rG4s from dG4s, and suppress *hTERC* rG4–nucleolin interactions (47). Furthermore, the L-aptamer, L-Apt.8f, is more specific for its intended *APP* rG4 target than for other rG4s, dG4s and non-G4s (48). However, when L-Apt.8f was tested on a panel of structural motifs, two off-targets were observed; specifically, L-Apt.8f showed cross-reactivity with *Kras1* rG4 and *Bcl2* rG4, albeit with slightly weaker binding affinities (48). L-Apt.8f has been applied to control *APP* reporter gene and native transcript gene expression in cells by targeting the *APP* rG4 structure (48). Overall, despite the improved specificity of this new L-RNA aptamer toolset, distinguishing one particular rG4 from others is still challenging, probably due to the high structural similarity of G4s.

In this study, we present an innovative approach to address this challenge by harnessing the power of multiple recognition modules. rG4s are structurally similar, but the rG4 flanking sequences are divergent. Therefore, one promising avenue to enhance specificity is the rational integration of an ASO complementary to the rG4 flanking sequence. Using *APP* rG4 and the general rG4 binder L-Apt.4–1c as an example, we designed a simple and robust chemical strategy to ligate the ASO DNA and L-Apt.4–1c through click chemistry to generate the first L-aptamer–ASO conjugate, L-Apt.4–1c-ASO15nt*_(APP)_*, able to specifically recognize the *APP* rG4 region *in vitro* and in cells. We also applied L-Apt.4–1c-ASO15nt*_(APP)_* to inhibit *APP* gene expression in cells and investigated the mechanisms by which L-Apt.4–1c-ASO15nt*_(APP)_* controls gene activity.

## Materials and methods

### Materials

L-Apt.4–1c_5′hexynyl was synthesized, purified by high-performance liquid chromatography (HPLC; ChemGenes) and verified by HPLC and mass spectrometry (ChemGenes) (Supplementary Figure S1). ASO DNA_3′Azide and ASO DNA_5′FAM-3′Azide were synthesized, purified by HPLC and verified by capillary electrophoresis and mass spectrometry (Integrated DNA Technologies) (Supplementary Figures S2–S5). Each of the ASO DNA oligonucleotide (10nt, 15nt, 20nt_DNA_3′Azide and 15nt_DNA-5′FAM-3′Azide) was prepared at the 100 nmol scale, with the actual yields found to be 27.4, 25.1, 49.5 and 24.8 nmol, respectively. The extinction coefficients of the ASO DNAs were found to be 103 500, 159 800, 211 300 and 180 760 L⋅mol^−1^⋅cm^−1^, respectively. To synthesize the oligonucleotide, modified phosphoramidite and phosphite triester were used. The phosphoramidite was modified with a dimethoxytrityl (DMT) group at the 5′ hydroxyl position. The phosphite triester was modified with a diisopropylamino group and a 2-cyanoethyl group. The DMT group and other protecting groups were removed by treating the oligonucleotide with a specialized deblocking reagent (not disclosed by IDT due to confidentiality). RHAU53 peptide (Supplementary Table S2) was synthesized by Synpeptide. DHX36 protein (Supplementary Table S2) was purchased from OriGene. pcDNA3.1–3 × Flag-C was purchased from Youbio; subsequently, the *DHX36* ORF was synthesized and inserted into pcDNA3.1–3 × Flag-C via the BamHI and XhoI restriction enzyme sites to generate *DHX36*-pcDNA3.1–3 × Flag-C by Youbio. Wild-type (wt) *APP* rG4 region-reporter insertion and mutated (mut) *APP* rG4 region-reporter insertion (Supplementary Table S1) were synthesized and inserted into psiCHECK-2 to generate wt *APP* rG4 region-psiCHECK-2 and mut *APP* rG4 region-psiCHECK-2 plasmids via the NotI restriction enzyme site by Genewiz. All sequences in plasmids were verified by Sanger sequencing.

### Methods

#### Click reaction

Eighty micromolar (μM) alkyne-modified aptamer (L-Apt.4–1c_5′hexynyl), 320 μM azide-modified oligonucleotides (ASO 10nt_DNA_3′Azide, ASO 15nt_DNA_3′Azide or ASO 20nt_DNA_3′Azide), 0.2 M triethylammonium acetate, freshly prepared 0.5 mM ascorbic acid, 0.5 mM Cu (II)–[Tris (benzyl triazolyl methyl) amine] (TBTA) and 45% dimethyl sulfoxide (*v/v*) were mixed and degassed by nitrogen gas. Then, a click reaction was performed in a thermoshaker (Hangzhou Allsheng Instruments, MSC-100) at 40°C for 4–6 h with shaking at 1300 rpm. After that, the products were resolved on 15% denaturing polyacrylamide gel (*w/v*) at 300 V for 40 min in 1× Tris-borate-ethylenediaminetetraacetic acid (TBE) buffer (pH = 8.3). Then, the product bands were cut out, and the gel was crushed and suspended in TEL800 buffer [1× Tris-ethylenediaminetetraacetic acid (TE) (pH = 8.0), 0.8 M LiCl] with shaking at 1300 rpm in a thermoshaker (Hangzhou Allsheng Instruments, MSC-100) overnight at 4°C. Then, the crushed gel was filtered with a 0.45 μm Spin-X centrifuge tube filter (Costar) and the conjugate was recovered by an RNA Clean & Concentrator-5 (Zymo Research) following the manufacturer’s instructions. To assess the purity of the conjugates, the products were resolved in 15% denaturing polyacrylamide gel (*w/v*), stained with SYBR Gold and scanned using a ChemiDoc Touch Imaging System (Bio-Rad). The conjugates were frozen at −30°C until use.

#### Matrix-assisted laser desorption ionization–time of flight mass spectrometry

To prepare the matrix-assisted laser desorption ionization–time of flight mass spectrometry (MALDI-TOF MS) matrix, ammonium citrate dibasic and 2′,6′-dihydroxyacetophenone were dissolved in 50% methanol (*v/v*) to saturation. Then, the conjugated oligonucleotides and matrix were mixed at a ratio of 1:1. After that, the samples were loaded onto the MALDI plate and dried in a fume hood. The plate was then loaded into a 4800 Plus MALDI TOF/TOF Analyzer (Applied Biosystems) for detection.

#### Electrophoretic mobility shift assay for binding assay

To perform electrophoretic mobility shift assay (EMSA), rG4 region/dG4 region/non-G4 motif oligonucleotides were fluorescently labeled and the aptamer/conjugate itself was not fluorescently labeled. For structure refolding, FAM/HEX_RNA/DNA oligonucleotides, L-aptamer or L-Apt.4–1c-ASO conjugates were heated at 95°C for 5 min, 25°C for 5 min and then 4°C for at least 30 min separately in buffer A [150 mM KCl, 1 mM MgCl_2_, 25 mM Tris-HCl (pH = 7.5)] supplemented with 8% sucrose (*w/v*). To calculate dissociation constants (*K*_d_) and achieve the pseudo-first-order condition, L-aptamers or conjugates were serially diluted from at least 100 nM before heating. After refolding, 10 nM RNA oligonucleotides and the indicated conjugates/L-aptamers were mixed and incubated for 30 min at 37°C for binding. Then, the samples were loaded into and resolved in 10% (19:1, acrylamide/bis-acrylamide) native polyacrylamide gel (*w/v*) for 70 min at 35 mA at 4°C in the running buffer B [50 mM KOAc, 25 mM Tris-HCl (pH = 7.5), 1 mM MgCl_2_]. Finally, the gels were scanned using the Typhoon laser-scanner platform (Cytiva). The intensity of the gel bands was analyzed by ImageJ software, followed by subtraction of background intensity. *K*_d_ was calculated with GraphPad Prism 8.2.1 (using the non-linear regression/binding-saturation/one site-specific binding model) by inputting XY values. Here, X is the concentration of serially diluted conjugates/aptamers/proteins, and Y is the bound fraction, obtained from the formula below. The band intensities of Bound or Unbound were obtained using ImageJ software with background intensity subtracted. Values were obtained from three replicates, and error bars display the standard error of the mean. *P* values were calculated using two-tailed *t*-tests, with *P* < 0.05 taken to indicate significant differences.


\begin{eqnarray*}{\mathrm{Fraction\ bound}} = \frac{{{\mathrm{Bound}}}}{{{\mathrm{Bound}} + {\mathrm{Unbound}}}}\end{eqnarray*}

#### EMSA for competition assay

To perform competition assay, rG4 region oligonucleotides were fluorescently labeled. L-Apt.4–1c-ASO15nt*_(APP)_* conjugate was serially diluted, and then the conjugate and the HEX_*APP* rG4 wt region were refolded by heating at 95°C for 5 min, 25°C for 5 min and then 4°C for at least 30 min separately in buffer A [150 mM KCl, 1 mM MgCl_2_, 25 mM Tris-HCl (pH = 7.5)] supplemented with 8% sucrose (*w/v*). Additionally, 48 nM DHX36 protein (or 100 nM RHAU53 peptide), 5 nM HEX_*APP* rG4 wt region/FAM_TRF2 rG4 region, and the indicated L-Apt.4–1c-ASO15nt*_(APP)_*/L-Apt.4–1c_5′hexynyl/ASO15nt_(APP)_ were mixed and incubated for 30 min at 37°C for competition binding. Then, the samples were loaded into and resolved in 6% (37.5:1, acrylamide/bis-acrylamide) native polyacrylamide gel (*w/v*) for 70 min at 35 mA at 4°C in the running buffer B [50 mM KOAc, 25 mM Tris-HCl (pH = 7.5), 1 mM MgCl_2_] (for DHX36) or at 12.5 mA for 75 min in the running buffer C [0.5× TBE (pH = 8.3) and 40 mM KOAc] (for RHAU53). Finally, the gels were scanned using the Typhoon laser-scanner platform (Cytiva). The intensity of the gel bands was analyzed by ImageJ software, followed by subtraction of background intensity. IC_50_ was calculated with GraphPad Prism 8.2.1 using the log[inhibitor] versus normalized response–variable slope model. X is the concentration of serially diluted conjugates, and Y (the bound fraction) is obtained from the formula below. Values were obtained from three replicates, and error bars display standard errors of the mean. *P* values were calculated using two-tailed *t*-tests, with *P* < 0.05 taken to indicate significant differences.


\begin{eqnarray*} &&{\mathrm{Fraction\ bound}} \nonumber \\ &&= \frac{{{\mathrm{rG}}4{\mathrm{\ }}\& {\mathrm{\ DHX}}36{\mathrm{\ complex}}}}{{{\mathrm{rG}}4{\mathrm{\ }}\& {\mathrm{\ conjugate\ complex}} + {\mathrm{rG}}4{\mathrm{\ }}\& {\mathrm{\ DHX}}36{\mathrm{\ complex}}}} \nonumber \\ && \times 100 \nonumber \\ \end{eqnarray*}

#### EMSA for unwinding assay

To perform unwinding assay, the *APP* rG4 region oligonucleotide was fluorescently labeled. For structure refolding, 10 nM FAM_*APP* rG4 region was subjected to a thermal denaturation step at 95°C for 5 min, followed by a slow cooling process to reach a temperature of 21°C at a ramp rate of 0.1°C/s in unwinding buffer [150 mM KCl, 2 mM MgCl_2_ and 25 mM Tris-HCl (pH = 7.5)]. For marker preparation, 100 nM of single-stranded RNA (ssRNA) trap was added before refolding, and 150 mM LiCl instead of KCl in buffer was used to facilitate annealing. Next, 0.11 μg of DHX36 protein was introduced into the system for 10 min for binding at 37°C. Then, 500 nM of ssRNA trap and 100 nM of adenosine triphosphate (ATP) or adenylyl-imidodiphosphate (AMP-PNP) (a non-hydrolysable analog of ATP) were introduced and incubated for 10 min at 37°C to promote unwinding. Then, 10 μg of proteinase K was added and incubated at 37°C for 40 min to digest the DHX36 protein. The total reaction volume was 12.5 μl. Afterwards, the samples were loaded into and resolved in 13% (19.5:1, acrylamide/bis-acrylamide) native polyacrylamide gel (*w/v*) for 80 min at 35 mA at 4°C in the running buffer C [0.5× TBE (pH = 8.3) and 40 mM KOAc]. Finally, the gels were scanned using the Typhoon laser-scanner platform (Cytiva).

#### Microscale thermophoresis

To perform microscale thermophoresis (MST), rG4 region oligonucleotides were fluorescently labeled. L-Apt.4–1c_5′hexynyl or L-Apt.4–1c-ASO conjugates were serially diluted from the maximum concentration for 15 times to generate 16 sets. Then, L-Apt.4–1c_5′hexynyl or conjugates and FAM_RNA oligonucleotides were subjected to thermal denaturation by heating to 95°C for 5 min, and then cooled to 25°C for 5 min and finally chilled to 4°C for at least 30 min in buffer A [150 mM KCl, 1 mM MgCl_2_, 25 mM Tris-HCl (pH = 7.5)], separately. After that, 30 nM FAM_RNA oligonucleotides were mixed with the diluted L-Apt.4–1c_5′hexynyl or conjugates and incubated at 37°C for 30 min. The samples were subsequently transferred into Nano-Temper Monolith NT.115 capillary tubes and detected using a Monolith NT.115 instrument in blue light mode. Finally, the data were analyzed using NanoTemper analysis software in the ‘Initial Fluorescence Analysis’ mode to determine the *K*_d_ values. The binding curves were directly plotted in GraphPad Prism 8.2.1 using the NanoTemper analysis software exported fit-values of the MST data.

#### Western blotting

HeLa cells (5–10 × 10^4/^well) were cultured and allowed to adhere onto a 24-well plate. Following a 24 h incubation period, transfection was performed to deliver the oligonucleotides (L-Apt.4–1c-ASO*_(APP)_* conjugates, L-Apt.4–1c_5′hexynyl, ASO DNA) into the cells using lipofectamine 2000. After transfection for the indicated time, the cells were collected and disrupted using lysis buffer D [50 mM Tris-HCl (pH = 7.5), 1% Triton X-100 (*v/v*), 250 mM NaCl and 5 mM ethylenediaminetetraacetic acid (EDTA)] supplemented with 1× proteinase inhibitor (Thermo Fisher Scientific). After that, the sample lysates were boiled with 1× Laemmli sample buffer (pH = 6.8) (Bio-Rad) and resolved by 8% sodium dodecyl sulfate–polyacrylamide gel electrophoresis (*w/v*). Western blotting was performed as described elsewhere (48). Briefly, the key steps involved gel running, transfer of proteins to membrane, blocking to avoid non-specific binding, primary antibody incubation, washing away unbound antibodies, secondary antibody incubation, further washing and the use of an enhanced chemiluminescence substrate for signal generation. Finally, the membranes were scanned by a ChemiDoc Touch Imaging System (Bio-Rad). The protein intensity was analyzed as adjusted volume (background subtracted) utilizing Image Lab software (Bio-Rad). Relative intensity of bands was calculated using the formula below. APP antibody (Millipore Sigma, MAB348) was diluted in 10% milk (*w/v*) (1:1000) and GAPDH antibody (Santa Cruz Biotechnology, sc-32233) was diluted in 5% milk (*w/v*) (1:1000). Values were obtained from three replicates, and error bars display the standard error of the mean. *P* values were calculated using two-tailed *t*-tests, with *P* < 0.05 taken to indicate significant differences.


\begin{eqnarray*}{\mathrm{Relative\ intensity}} = \frac{{{\mathrm{adjusted\ volume\ of\ APP}}}}{{{\mathrm{adjusted\ volume\ of\ GAPDH}}}}\end{eqnarray*}

#### Dual luciferase reporter gene assay

HeLa cells (2 × 10^4/^well) were cultured and allowed to adhere onto a 96-well plate. Following a 24 h period, 50 ng wt *APP* rG4 region-psiCHECK-2 (or mut *APP* rG4 region-psiCHECK-2) plasmid and 40 nM (or 0 nM) L-Apt.4–1c-ASO15nt*_(APP)_* (L-Apt.4–1c_5′hexynyl or ASO15nt*_(APP)_*) were sequentially transfected into cells using lipofectamine 2000. After about 24 h, the Renilla and Firefly luciferase activities were measured using a Dual-Luciferase Reporter Assay System (Promega), following the manufacturer's instructions, in a SpectraMax iD5 Multi-Mode Microplate Reader (Molecular Devices). Before transfection, L-Apt.4–1c-ASO15nt_*(APP)*_ (L-Apt.4–1c_5′hexynyl or ASO15nt*_(APP)_*) was heated at 95°C for 5 min, 25°C for 5 min and then 4°C for at least 30 min separately in buffer A [150 mM KCl, 1 mM MgCl_2_, 25 mM Tris-HCl (pH = 7.5)]. Luciferase activity was calculated using the formula below.


\begin{eqnarray*}{\mathrm{Normalized\ luciferase\ activity}} = \frac{{{\mathrm{Renilla}}}}{{{\mathrm{Firefly}}}}\end{eqnarray*}

#### Cell imaging

RNAs containing the rG4 sequence were produced through *in vitro* transcription by a T7 High Yield RNA Synthesis Kit (NEB) following the manufacturer's instructions. Briefly, T7-imaging-forward DNA strands and imaging-reverse DNA strands (Supplementary Table S1) were hybridized in 10 μM NaCl by incubating at 95°C for 5 min and then 4°C for several hours, to form the DNA template for transcription. Then, transcription was conducted at 37°C for 3.5 h, and the DNA template was digested by Turbo DNase. After resolving by 12% denaturing polyacrylamide gel (*w/v*), the RNAs were crushed and suspended in TEL800 buffer [1× TE (pH = 8.0), 0.8 M LiCl] with shaking in a thermoshaker (Hangzhou Allsheng Instruments, MSC-100) at 1300 rpm overnight at 4°C. Next day, the crushed gel was filtered with a 0.45 μm Spin-X centrifuge tube filter (Costar) and the RNAs were recovered from the gel using RNA Clean & Concentrator-5 (Zymo Research) following the manufacturer's instructions. HeLa cells (2.5 × 10^5^) were cultured and allowed to adhere onto 35 mm confocal dishes. Following a 24 h incubation period, the HeLa cells were transfected with 50 nM refolded *in vitro* transcribed RNAs in buffer A [150 mM KCl, 1 mM MgCl_2_, 25 mM Tris-HCl (pH = 7.5)] by lipofectamine 2000. After at least 7 h transfection, the cells underwent fixation through treatment with 4% paraformaldehyde (*w/v*) for 15 min at 25°C. Subsequently, the cell membranes were permeabilized by exposure to 0.5% Triton X-100 (*v/v*) for 30 min at 37°C, and washed with 2× saline–sodium citrate (SSC) (pH = 7.0). FAM_L-Apt.4–1c-ASO15nt_*(APP)*_ was refolded by heating at 95°C for 5 min, 25°C for 5 min and then 4°C for at least 30 min in buffer A [150 mM KCl, 1 mM MgCl_2_, 25 mM Tris-HCl (pH = 7.5)]. Then, the cells were stained with 0.2 μM Cy3-probes and 15 nM refolded FAM_L-Apt.4–1c-ASO15nt*_(APP)_* sequentially overnight at 37°C, followed by dilution in staining buffer [4× SSC (pH = 7.0), 30% deionized formamide (*v/v*), 10% dextran sulfate (*w/v*), 0.5 mM EDTA]. After washing with 2× SSC (pH = 7.0) five times, the cells were exposed to a 5 μg/ml solution of Hoechst 33342 dye for a 15 min incubation period at 37°C and scanned by confocal microscopy (Leica SPE) with a 63× oil objective. The setting was as follows: for FAM (λ_ex_= 488 nm, λ_em_= 510–545 nm), for Cy3 (λ_ex_= 532 nm, λ_em_= 560–605 nm), for Hoechst 33342 (λ_ex_= 405 nm, λ_em_= 430–477 nm). At least 350 cells for each group set were scanned.

For RNase H1 subcellular distribution detection, 2.5 × 10^5^ HeLa cells were cultured and allowed to adhere onto 35 mm confocal dishes for 24 h. Then, the cells underwent fixation through treatment with 4% paraformaldehyde (*w/v*) for 15 min at 25°C. Subsequently, the cell membranes were exposed to 0.3% Triton X-100 (v/v) for 10 min at 25°C for permeabilization. Then, the cells were washed with phosphate-buffered saline (PBS) (pH = 7.4) and blocked with 1% bovine serum albumin (BSA) (*w/v*) in PBS (pH = 7.4) for 30 min at 25°C. RNase H1 primary antibody [Proteintech 15606–1-AP, 1:100 in 1% BSA (*w/v*)] was incubated with the cells at 4°C. After overnight incubation, the cells were subjected to five rounds of washing with PBS (pH = 7.4) and treated with Alexa Fluor^®^ 555 secondary antibody [Abcam ab150078, 1:500 in 1% BSA (*w/v*)] at 25°C for 1 h. Then, after washing with PBS (pH = 7.4) five times, and staining with Hoechst 33342 (5 μg/ml) for 20 min at 37°C, the cells were scanned by confocal microscopy (Leica SPE) with a 63× oil objective. The setting was as follows: for RNase H1 (λ_ex_= 532 nm, λ_em_= 550–600 nm), for Hoechst 33342 (λ_ex_= 405 nm, λ_em_= 420–500 nm). About 150 cells were scanned.

#### RNase H cleavage assay

HEX_*APP* rG4 wt region and L-Apt.4–1c-ASO15nt*_(APP)_* were first refolded by heating at 95°C for 5 min, 25°C for 5 min and then 4°C for at least 5 min independently in buffer A [150 mM KCl, 1 mM MgCl_2_, 25 mM Tris-HCl (pH = 7.5)]. A mixture of HEX_*APP* rG4 wt region (300 nM) and L-Apt.4–1c-ASO15nt_*(APP)*_ was prepared and incubated at 37°C for 10 min in 1× RNase H Reaction Buffer [50 mM Tris-HCl (pH = 8.3), 75 mM KCl, 3 mM MgCl_2_ and 10 mM DTT]. After that, the indicated amount of RNase H was added to react at 37°C. At the indicated time, 25 mM EDTA and an equal volume of formamide were added to stop the reaction. Next, prior to separation, the samples were denatured by incubating them at 95°C for 3 min, and subsequently resolved on urea-containing 15% denaturing polyacrylamide gel (*w/v*). Finally, the gels were scanned using a Typhoon laser-scanner platform (Cytiva).

#### Reverse transcription–quantitative polymerase chain reaction

HeLa cells (9 × 10^4^/well) were cultured and allowed to adhere onto a 24-well plate. Following a 24 h incubation period, L-Apt.4–1c-ASO*_(APP)_*, L-Apt.4–1c_5′hexynyl or ASO*_(APP)_* was transfected into the cells by lipofectamine 2000. After harvesting the HeLa cells, RNAs were isolated using a MiniBEST Universal RNA Extraction Kit (TaKaRa), in accordance with the step-by-step instructions provided by the manufacturer. Then, total RNAs (100–150 ng) were reverse transcribed into cDNA utilizing a PrimeScript reverse transcription (RT) reagent kit (Perfect Real Time; Takara RR037Q), and about 100 ng cDNA, 0.5 μM forward primer, 0.5 μM reverse primer and 1 × SsoAdvanced Universal SYBR Green Supermix (Bio-Rad) were mixed in a 10 μl reaction vessel. Subsequently, the mixture was subjected to quantitative polymerase chain reaction (qPCR) analysis using a CFX Connect real-time system (Bio-Rad) under the following protocol: denaturation 98°C 30 s, (denaturation 98°C 10 s, annealing/extension 60°C 30 s, total 40 cycles), melt curve analysis: 65–95°C in 0.5°C increments at 5 s/step. To increase the accuracy of the experiments, APP primers at different locations (*APP* and *APP′*) were designed and two housekeeping genes (*GAPDH* and *18S rRNA*) were used as internal references (Supplementary Table S1). The primers were designed with PrimerBank or Primer-BLAST. Finally, the relative mRNA level of *APP* was calculated in Microsoft Excel using the formula below. Values were obtained from three replicates, and error bars display the standard error of the mean. *P* values were calculated using two-tailed *t*-tests, with *P* < 0.05 considered to indicate significant differences.


\begin{eqnarray*}{\mathrm{Relative\ mRNA\ level\ of\ }}APP = {{2}^{ - \ \left( {{\mathrm{\Delta \Delta Cq}}} \right)}}\end{eqnarray*}

\begin{eqnarray*}{\mathrm{\Delta Cq}} = {\mathrm{Cq}}\ \left( {APP} \right)\ -{\mathrm{\ Cq}}\ \left( {housekeeping\ gene} \right)\end{eqnarray*}

\begin{eqnarray*} \mathrm{\Delta \Delta Cq} &=& {\mathrm{\Delta Cq\ }}\left( {{\mathrm{conjugate\ or\ aptamer}}} \right){\mathrm{\ }} \nonumber \\ && -{\mathrm{\ \Delta Cq\ }}\left( {{\mathrm{without\ conjugate\ or\ aptamer}}} \right)\end{eqnarray*}

#### RNA immunoprecipitation

HeLa cells (13.5 × 10^6^) were lysed in 1 ml Radioimmunoprecipitation assay (RIPA) buffer (pH = 7.6) (Thermo Fisher Scientific) supplemented with 1× proteinase inhibitor (Thermo Fisher Scientific) and 200 U/ml Ribolock (Thermo Fisher Scientific). Eighty microliters of streptavidin magnetic beads (MedChemExpress) were washed with wash buffer I [10 mM Tris-HCl (pH = 7.5), 1 mM EDTA, 1 M KCl, 0.05% Tween-20 (*v/v*)], three times and blocked with 5 mg/ml BSA and 0.2 mg/ml transfer RNA for 1 h. For structure refolding, 100 pmol Biotin_wt *APP* rG4 region (or Biotin_mut *APP* rG4 region) oligonucleotide (Supplementary Table S1) was heated at 95°C for 5 min, 25°C for 5 min and then 4°C for at least 5 min separately in buffer E [150 mM KCl, 0.1 mM EDTA, 25 mM Tris-HCl (pH = 7.5)]. L-Apt.4–1c-ASO15nt*_(APP)_* was heated at 95°C for 5 min, 25°C for 5 min and then 4°C for at least 5 min in buffer A [150 mM KCl, 1 mM MgCl_2_, 25 mM Tris-HCl (pH = 7.5)]. Then, an equal amount of the blocked streptavidin magnetic beads was incubated with the refolded Biotin_wt (or mut) *APP* rG4 region oligonucleotide separately for 1 h at 23°C. After that, the beads were washed with buffer E three times. Seventy microliters of cell lysate was diluted with buffer A to 200 μl and the beads were incubated with the diluted cell lysate with/without an additional 100 nM L-Apt.4–1c-ASO15nt_*(APP)*_ for about 2 h at 4°C. Then, the beads were washed with buffer A two times and buffer A supplemented with 100 mM NaCl or 200 mM NaCl, sequentially. Then, the beads were boiled with 1× Laemmli sample buffer (pH = 6.8) (Bio-Rad) at 95°C for 10 min for elution. Finally, the eluted samples were subjected to western blotting detection with DHX36 antibody (Thermo Fisher Scientific, PA5-78644) in 5% milk (*w/v*) (1:1000). Three replicates were performed.

## Results

### Design of a novel L-aptamer–ASO conjugate that can recognize a particular rG4 region of interest

To increase the specificity of the L-aptamer, we devised a novel strategy to synthesize conjugates, named L-aptamer–ASO, to target a particular rG4 region of interest. L-aptamer–ASO is composed of two modules: (1) an L-aptamer for the recognition of the rG4 motif via structural recognition, and (2) an antisense DNA oligonucleotide (ASO sequence) for the recognition of the flanking sequence next to the rG4 motif via base pair hybridization (Figure 1B). Here, as we aimed to use L-Apt.4–1c to target *APP* rG4, we assembled L-Apt.4–1c with the ASO sequence reverse complementarily to the 3′-flanking sequence of the *APP* rG4 structure. To covalently connect the two modules, the 5′-end of L-Apt.4–1c was modified with hexynyl and the 3′-end of the ASO DNA was modified with azide. Then, through a click chemistry reaction in the presence of ascorbic acid and Cu (II)–TBTA, L-Apt.4–1c and ASO DNA were synthetically linked to produce the L-aptamer–ASO conjugate, namely L-Apt.4–1c-ASO (Figure 1C).

### Synthesis and characterization of L-aptamer–ASO conjugates that enhance binding affinity to *APP* rG4 region

In this study, we target a well-studied rG4, *APP* rG4, which is located in the 3′ UTR of *APP* mRNA. In our previous study, L-Apt.4–1c was found to preferentially interact with a number of rG4s over dG4s and non-G4 DNA/RNA structures (47,51). To verify that L-Apt.4–1c can bind to *APP* rG4, we conducted an EMSA, in which strong binding of the rG4 and L-aptamer would lead to a new band with decreased electrophoretic mobility on the gel. The EMSA result showed that L-Apt.4–1c can indeed bind to the *APP* rG4 motif, with a *K*_d_ value 26.3 ± 2.6 nM (Supplementary Figure S6). To determine whether the hexynyl group in L-Apt.4–1c affects the binding to the *APP* rG4 motif, we tested the binding between the *APP* rG4 wt motif and L-Apt.4–1c_5′hexynyl. The EMSA result showed that hexynyl had almost no effect on binding (*K*_d_ = 30.1 ± 1.6 nM) (Supplementary Figure S7), suggesting that it could be used for the subsequent click reaction without impacting the binding affinity to the target of interest. In addition, we considered whether the flanking sequence of *APP* rG4 affects recognition by the L-aptamer, and therefore we determined the binding between the *APP* rG4 wt region (*APP* rG4 motif with flanking sequence) and L-Apt.4–1c_5′hexynyl. The EMSA result showed that introduction of the flanking sequence only affected the binding slightly, with *K*_d_ = 60.4 ± 2.6 nM (Supplementary Figure S7). We reasoned that this was likely due to the competitive, alternative RNA secondary structures that can form within the sequence. Lastly, to verify that the binding between L-Apt.4–1c_5′hexynyl and *APP* rG4 was rG4 structure-dependent, the middle Gs of the G-tracts in *APP* rG4 were mutated to A, to disrupt rG4 formation in the construct (38). The EMSA results showed that L-Apt.4–1c_5′hexynyl bound neither the *APP* rG4 mut motif nor the *APP* rG4 mut region (Supplementary Figure S7), indicating that the binding is dependent on the rG4 structure.

**Figure 2.**
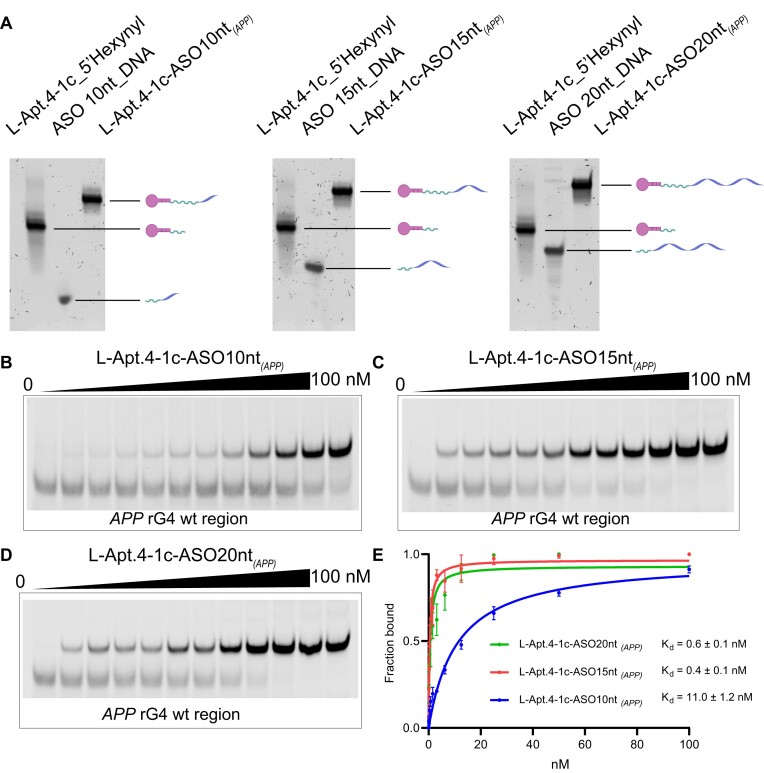
L-Apt.4–1c-ASO conjugates show enhanced binding affinity to *APP* D-rG4 region. (**A**) The synthesis of L-Apt.4–1c-ASO*_(APP)_* conjugates. Different lengths of ASO (10, 15 and 20 nt) that are reverse complementary to the *APP* transcript were designed. They are referred to as L-Apt.4–1c-ASO10nt_*(APP)*_, L-Apt.4–1c-ASO15nt_*(APP)*_ and L-Apt.4–1c-ASO20nt_*(APP)*_, respectively. From the denaturing polyacrylamide gel, a new band with decreased electrophoretic mobility was observed after reaction, suggesting the successful chemical ligation of L-aptamer and ASO. (**B**) Binding of L-Apt.4–1c-ASO10nt_*(APP)*_ against *APP* rG4 wt region analyzed by EMSA. (**C**) Binding of L-Apt.4–1c-ASO15nt_*(APP)*_ against *APP* rG4 wt region analyzed by EMSA. (**D**) Binding of L-Apt.4–1c-ASO20nt_*(APP)*_ against *APP* rG4 wt region analyzed by EMSA. (**E**) Binding curve of L-Apt.4–1c-ASO10nt_*(APP)*_, L-Apt.4–1c-ASO15nt_*(APP)*_ and L-Apt.4–1c-ASO20nt_*(APP)*_ against *APP* rG4 wt region from the EMSA results in panels (**B–D**). The *K*_d_s for L-Apt.4–1c-ASO10nt_*(APP)*_, L-Apt.4–1c-ASO15nt*_(APP)_* and L-Apt.4–1c-ASO20nt_*(APP)*_ were determined to be 11.0 ± 1.2 nM, 0.4 ± 0.1 nM and 0.6 ± 0.1 nM, respectively. Compared with L-Apt.4–1c (*K*_d_ = 60.4 ± 2.6 nM), L-Apt.-4–1c-ASO_*(APP)*_ conjugates bound to the *APP* rG4 wt region more strongly with lower *K*_d_ values. Values were obtained from three replicates and error bars display standard errors of the mean. *P* values were calculated using two-tailed *t*-tests with *P* < 0.05 considered to indicate significant differences. The HEX_*APP* rG4 wt region used had a concentration of 10 nM.

After confirming the *APP* rG4 as the target, we designed the ASO DNA sequence complementarily to the flanking sequence of the *APP* rG4 motif. For the following reasons, we chose DNA as the complementary ASO sequence. (1) Commercial azide-modified DNA is easy and inexpensive to obtain. (2) Hybridized double-stranded DNA and RNA can likely recruit RNase H in cells to digest the RNA in DNA:RNA hybrids, thereby potentially enhancing the inhibition efficacy of the conjugate against gene expression. Subsequently, through the click reaction, we obtained the conjugate product, which we gel-purified before use (Supplementary Figure S8). To investigate whether the length of the ASO DNA sequence could affect binding, we designed a set of lengths of ASO i.e. 10, 15 and 20 nt. The corresponding conjugates are referred to as L-Apt.4–1c-ASO10nt_(APP)_, L-Apt.4–1c-ASO15nt_(APP)_ and L-Apt.4–1c-ASO20nt_(APP)_*_,_* respectively. In the denaturing polyacrylamide gel, the ASO DNA or conjugates of all three sizes showed the correct relative positions, with larger ones having lower electrophoretic mobility (Figure 2A). Furthermore, MALDI-TOF-MS verified that the molecular weight of the conjugates was correct (Supplementary Figures S9–S11), together indicating the successful synthesis of the L-Apt.4–1c-ASO conjugates.

After the synthesis of the conjugates, we measured the binding affinity to the *APP* rG4 wt region using EMSA to determine whether the conjugates improved the binding. As expected, L-Apt.4–1c-ASO10nt_*(APP)*_, L-Apt.4–1c-ASO15nt*_(APP)_* and L-Apt.4–1c-ASO20nt*_(APP)_* all significantly improved target binding compared with the L-Apt.4–1c-5′ hexynyl set, with *K*_d_ values changing from 60.4 ± 2.6 nM to 11.0 ± 1.2 nM, 0.4 ± 0.1 nM and 0.6 ± 0.1 nM respectively (Supplementary Figure S7 and Figure 2B–E). We also determined the binding between the three L-Apt.4–1c-ASO conjugates and the *APP* rG4 mut region. The EMSA results showed that the conjugates’ binding to the *APP* rG4 mut region was much weaker than to the *APP* rG4 wt region (Supplementary Figure S12), further supporting the importance of the rG4 structure recognition mode. Among the conjugates, L-Apt.4–1c-ASO15nt_*(APP)*_ and L-Apt.4–1c-ASO20nt_*(APP)*_ performed the best. Considering that their binding capabilities are similar, we chose L-Apt.4–1c-ASO15nt*_(APP)_* for subsequent experiments.

### L-aptamer–ASO conjugate specifically recognizes a particular rG4 region of interest both *in vitro* and in cells

**Figure 3.**
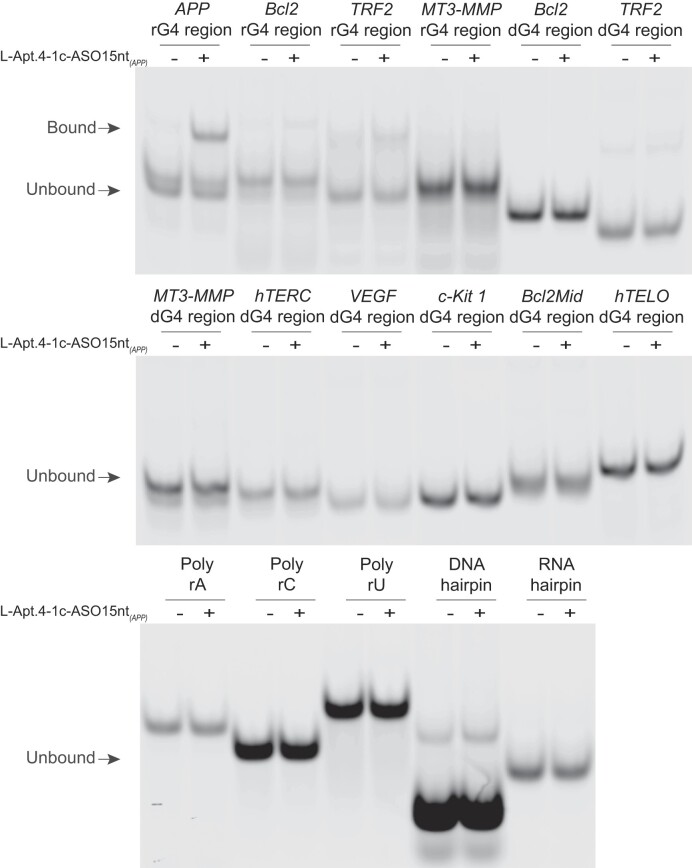
L-Apt.4–1c-ASO15nt*_(APP)_* conjugate displays strong binding affinity and specificity toward the *APP* rG4 wt region over other non-targets. Strong binding is observed between L-Apt.4–1c-ASO15nt*_(APP)_* and *APP* rG4 wt region. Weak binding is observed between L-Apt.4–1c-ASO15nt*_(APP)_* and *Bcl2* rG4 region, *TRF2* rG4 region and *MT3-MMP* rG4 region. No binding is observed between L-Apt.4–1c-ASO15nt*_(APP)_* and dG4 regions, namely *Bcl2* dG4 region, *TRF2 d*G4 region, *MT3-MMP* dG4 region, *hTERC* dG4 region, *VEGF* dG4 region, *c-Kit 1* dG4 region, *Bcl2Mid* dG4 region or *hTELO* dG4 region. No binding is observed between L-Apt.4–1c-ASO15nt*_(APP)_* and non-G4 motifs, including DNA and RNA hairpins and single-stranded poly rA/rC/rU RNAs. L-Apt.4–1c-ASO15nt*_(APP)_* preferentially interacts with *APP* rG4 wt region over other constructs tested. All the FAM_rG4 regions, FAM_dG4 regions and FAM_non-G4 motifs used had a concentration of 10 nM, and the L-Apt.4–1c-ASO15nt*_(APP)_* conjugate had a concentration of 5 nM.

To verify whether the conjugate indeed improved the specificity of target recognition, we selected several rG4s, including *Bcl2* rG4, *TRF2* rG4 and *MT3-MMP* rG4, which were previously reported to bind to L-Apt.4–1c (47), as controls for comparison. The EMSA result showed that L-Apt.4–1c-ASO15nt*_(APP)_* bound strongly to the *APP* rG4 region, while showing almost no binding to *Bcl2* rG4, *TRF2* rG4 and *MT3-MMP* rG4 regions, indicating an improvement in binding specificity (Figure 3). Furthermore, we compared the binding capacity of the *Bcl2* rG4 region, *TRF2* rG4 region and *MT3-MMP* rG4 region to L-Apt.4–1c_5′hexynyl or L-Apt.4–1c-ASO15nt_*(APP)*_ using EMSA and calculated the *K*_d_ values to be 128.2 ± 23.3 nM and 319.9 ± 53.1 nM, 39.0 ± 4.9 nM and 34.1 ± 6.8 nM and 5.4 ± 3.4 μM and 7.1 ± 5.3 μM, respectively (Supplementary Figure S13). Unlike the significantly enhanced binding to the *APP* rG4 region (Figure 2 and Supplementary Figure S7B and D), L-Apt.4–1c-ASO15nt*_(APP)_* showed no clear pattern for enhanced binding to other non-target rG4 regions using L-Apt.4–1c_5′hexynyl as a comparison reference (Supplementary Figure S13). The affinity data obtained with EMSA (Supplementary Figure S13) were further verified by MST, which showed a similar result (Supplementary Figure S14). In addition, we determined the binding between L-Apt.4–1c-ASO15nt_*(APP)*_ and a few dG4s regions, including the *Bcl2* dG4 region, *TRF2 d*G4 region, *MT3-MMP* dG4 region, *hTERC* dG4 region, *VEGF* dG4 region, *c-Kit 1* dG4 region, *Bcl2Mid* dG4 region and *hTELO* dG4 region and none of them bound to L-Apt.4–1c-ASO15nt*_(APP)_* (Figure 3). To investigate the impact of ASO on target binding, we conducted the binding of L-Apt.4–1c-ASO15nt_*(APP)*_ to non-G4 structural motifs, including DNA*/*RNA hairpin and poly A*/*C*/*U RNAs, as well as dG4 sequences, and none of them showed binding to L-Apt.4–1c-ASO15nt_*(APP)*_ (Figure 3), which revealed that the additional ASO sequence introduced into L-Apt.4–1c-ASO did not produce new binding sites for non-target structure recognition. Taken together, the novel L-aptamer–D-oligonucleotide tool enables the specific recognition of a single rG4 region (*APP* rG4) *in vitro*.

**Figure 4.**
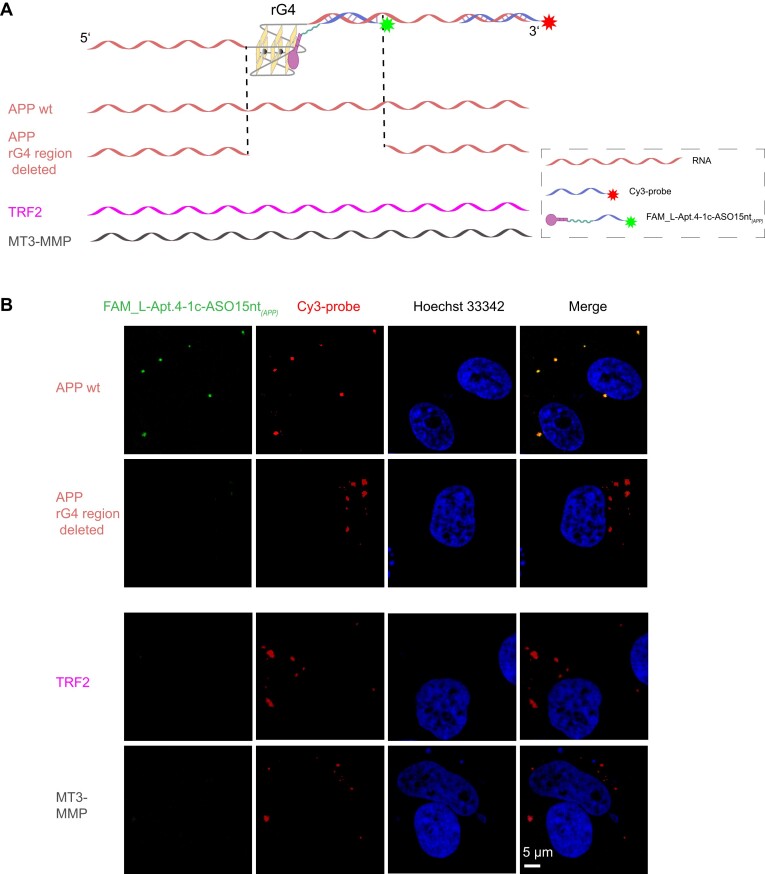
L-Apt.4–1c-ASO15nt*_(APP)_* conjugate preferentially colocalizes with the *APP* rG4-containing transcript over other rG4-containing transcripts in cells. (**A**) Schematic illustration of the cell-imaging assay. The RNA constructs (*APP* rG4 wt region, *APP* rG4 and flanking region deleted, *TRF2* rG4 region, *MT3-MMP* rG4 region) were transfected into HeLa cells independently. After cell membrane permeabilization induced by Triton X-100, Cy3-probe and FAM_L-Apt.4–1c-ASO15nt*_(APP)_* were used to stain the RNA and rG4 region separately. The Cy3-containing DNA probe was used to identify the location of the transfected RNA construct. The L-Apt.4–1c-ASO15nt*_(APP)_* was labeled with FAM to identify its colocalization with the corresponding RNA construct in cells. (**B**) Confocal microscopy of cells that were transfected with different RNA constructs, followed by staining with FAM-labeled L-Apt.4–1c-ASO15nt*_(APP)_* conjugate and Cy3-containing DNA probe. Nuclei were stained with Hoechst 33342. Only *APP* rG4 wt region showed colocalization with L-Apt.4–1c-ASO15nt*_(APP)_*.

Observing its strong *in vitro* binding specificity, we were encouraged to explore whether this new rG4 targeting tool can be applied to recognize a particular rG4 structure of interest in cells. We first *in vitro* transcribed the RNAs containing the rG4 region sequences and then transfected them into HeLa cells. In addition, to monitor the intracellular distribution of transfected RNA, a Cy3-labeled DNA oligonucleotide (Cy3 probe) complementary to the 3′ end of the RNA was used to hybridize and localize the RNA (Figure 4A). To obtain a fluorescently labeled conjugate, FAM-labeled ASO and L-Apt.4–1c_5′hexynyl were ligated to generate FAM_L-Apt.4–1c-ASO15nt*_(APP)_* through a click reaction (Supplementary Figure S15). After that, fixed and permeabilized HeLa cells were stained with FAM_L-Apt.4–1c-ASO15nt*_(APP)_* to visualize the *APP* rG4 region. To demonstrate that FAM_L-Apt.4–1c-ASO15nt*_(APP)_* bound to the *APP* rG4 region, we also set up *APP* rG4 and flanking sequence-deleted RNAs (*APP* rG4 region deleted) as controls (Figure 4A). As shown in Figure 4B, green FAM fluorescent foci were observed only in the *APP* wt group, not in the *APP* rG4 region-deleted group, and the FAM and Cy3 fluorescent foci were co-localized well, indicating that FAM_L-Apt.4–1c-ASO15nt*_(APP)_* correctly recognizes the *APP* rG4 region. In addition, to reveal the specificity of FAM_L-Apt.4–1c-ASO15nt*_(APP)_* in cells, we also tested the non-target rG4 region, including *TRF2* and *MT3-MMP*, as negative controls. Confocal imaging showed that only the red Cy3 signal of the RNA was detected in the non-target group, while no green FAM_L-Apt.4–1c-ASO15nt*_(APP)_* signal was detected at all and there were no colocalization foci, collectively suggesting specific recognition of the *APP* rG4 region (Figure 4B). To consolidate the interaction between the *APP* rG4 region and L-Apt.4–1c-ASO15nt*_(APP)_*, we also performed dual luciferase reporter gene assay by inserting the wt *APP* rG4 region-reporter insertion sequence (Supplementary Table S1) into the 3′UTR of the Renilla luciferase of psiCHECK-2 (wt *APP* rG4 region-psiCHECK-2) and used mut *APP* rG4 region-psiCHECK-2 as a control. Similar to previous studies (38,48), the normalized luciferase (Renilla/Firefly) activity of the wt group was lower than that of the mut group (Supplementary Figure S16). When treated with L-Apt.4–1c-ASO15nt*_(APP)_*, the normalized luciferase activity of the wt group decreased significantly compared with the non-treatment group (control). The treatment of L-Apt.4–1c_5′hexynyl also caused a significant decrease in the normalized luciferase activity of wt group, but the inhibition effect was weaker than that of L-Apt.4–1c-ASO15nt*_(APP)_*. Meanwhile, ASO had no significant inhibitory effect on the wt group (Supplementary Figure S16A). For the mut group, none of the treatments inhibited the luciferase activities (Supplementary Figure S16B). Together, the dual luciferase reporter assay further confirmed the direct interaction between the L-Apt.4–1c-ASO15nt*_(APP)_*. and *APP* rG4 region and the function of gene regulation. Overall, these results confirmed that the FAM_L-Apt.4–1c-ASO conjugate can selectively recognize a specific rG4 region of interest in cells.

### L-Apt.4-1c-ASO15nt*_(APP)_* can inhibit endogenous APP expression in cells through translation inhibition and RNase H-mediated knockdown of mRNA

**Figure 5.**
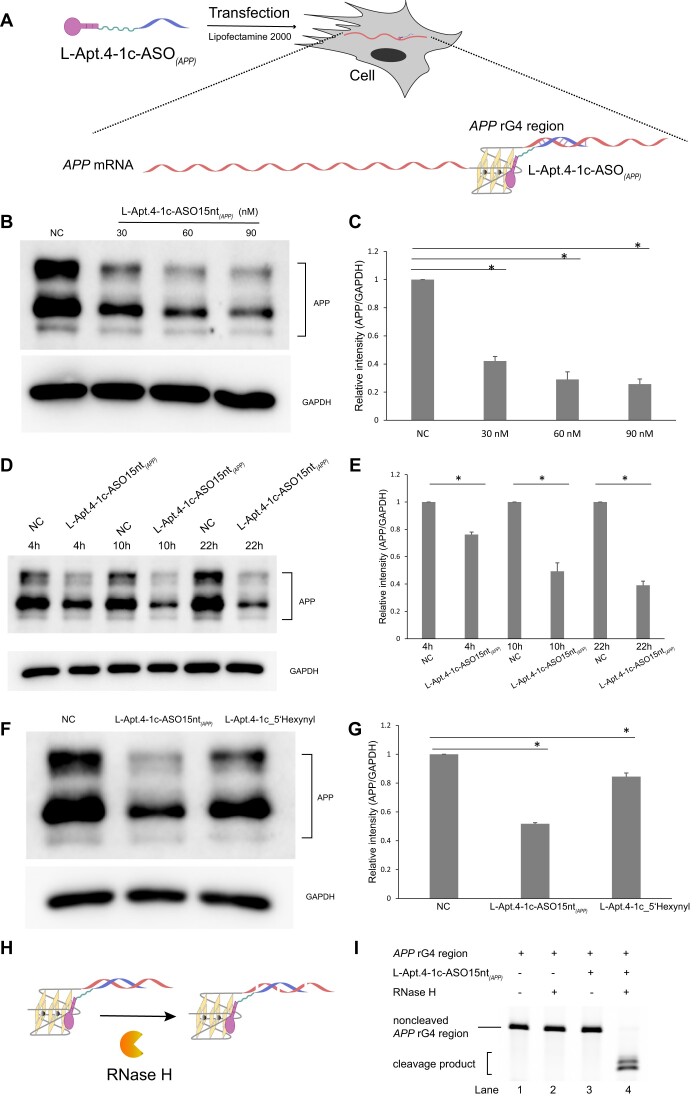
L-Apt.4–1c-ASO15nt*_(APP)_* conjugate inhibits endogenous APP protein in cells. (**A**) Schematic illustration of cell treatment with L-Apt.4–1c-ASO15nt*_(APP)_*. (**B**) Effect of different concentrations (0, 30, 60, and 90 nM) of L-Apt.4–1c-ASO15nt*_(APP)_* addition on APP expression in HeLa cells. The APP expression decreased with increasing L-Apt.4–1c-ASO15nt*_(APP)_* concentration. GAPDH was used as loading control. Cells were treated for 22 h. (**C**) Normalized APP expression results from western blotting obtained from (**B**). (**D**) Kinetic experiments (4, 10 and 22 h) of L-Apt.4–1c-ASO15nt*_(APP)_* addition on APP expression in HeLa cells. The APP expression decreased with increasing time of treatment. The concentration of L-Apt.4–1c-ASO15nt*_(APP)_* was 60 nM. (**E**) Normalized APP expression results from western blotting obtained from (**D**). (**F**) Comparison between L-Apt.4–1c-ASO15nt*_(APP)_* and L-Apt.4–1c_5′hexynyl. The APP expression of both was decrease, with L-Apt.4–1c-ASO15nt*_(APP)_* having a stronger inhibitory effect. (**G**) Normalized APP expression results from western blotting obtained from (**F**). (**H**) Schematic illustration of RNase H treatment assay *in vitro*. (**I**) RNase H treatment assay detected by denaturing polyacrylamide gel. RNase H cleaved the conjugate–*APP* rG4 region complex. Results were obtained from three replicates. Error bars represent the standard error of the mean. *P* values were calculated using two-tailed *t*-tests, with *P* < 0.05 considered to indicate significant differences.

Having shown that the conjugate can specifically recognize the *APP* rG4 region, we attempted to determine whether the conjugate can be applied to control endogenous *APP* gene expression by specifically targeting the *APP* rG4 region. To deliver L-Apt.4–1c-ASO15nt_*(APP)*_ into cells, Lipofectamine 2000 was used for transfection (Figure 5A) and the APP expression level was determined by western blotting. First, we tested the effect of different ASO length-conjugates (L-Apt.4–1c-ASO10nt_*(APP)*_, L-Apt.4–1c-ASO15nt_*(APP)*_ and L-Apt.4–1c-ASO20nt_*(APP)*_) on gene expression. The results showed that they all could be transfected into cells and inhibited APP protein expression (Supplementary Figure S17). Among the conjugates, L-Apt.4–1c-ASO15nt_*(APP)*_ had a stronger inhibitory effect than L-Apt.4–1c-ASO10nt_*(APP)*_, while L-Apt.4–1c-ASO15nt_*(APP)*_ and L-Apt.4–1c-ASO20nt*_(APP)_* showed similar inhibitory effects (Supplementary Figure S17), which again encouraged us to use the 15nt_conjugate for subsequent experiments. Second, after confirming the use of L-Apt.4–1c-ASO15nt_*(APP)*_, we treated cells with different concentrations of L-Apt.4–1c-ASO15nt_*(APP)*_ (0 nM (NC), 30 nM, 60 nM and 90 nM), and detected the APP protein level. As shown in Figure 5B and C, with increasing concentration of L-Apt.4–1c-ASO15nt_*(APP)*_, the APP protein levels decreased accordingly, meaning the inhibition was concentration-dependent. We also conducted kinetic experiments with L-Apt.4–1c-ASO15nt*_(APP)_* treatment for 4 h, 10 h, and 22 h separately. Compared with the NC group (Lipofectamine 2000-treated), at 4 h the inhibition of L-Apt.4–1c-ASO15nt*_(APP)_* was already significant. With increasing treatment time, the inhibitory effect increased and peaked at 22 h (Figure 5D and 5E), suggesting time-dependent inhibition. Third, to better understand the inhibitory mechanism of the conjugate, we compared the effects of L-Apt.4–1c-ASO15nt_*(APP)*_ and L-Apt.4–1c_5′hexynyl, using L-Apt.4–1c_5′hexynyl without ASO as negative control. The results showed that both of them inhibited the APP protein level significantly, but the effect of L-Apt.4–1c_5′hexynyl was weaker than that of L-Apt.4–1c-ASO15nt_*(APP)*_ (Figure 5F and 5G), which may be due to the stronger binding affinity of the latter to the *APP* rG4 region (Figure 2 and S7). As RNA:DNA hybrids can trigger RNase H cleavage of the RNA, we also hypothesized that one possible mechanism underlying the inhibitory effect of L-Apt.4–1c-ASO15nt*_(APP)_* was RNase H-mediated degradation of RNA. To verify this, we performed RT-qPCR to measure the *APP* mRNA levels after treatment with L-Apt.4–1c-ASO15nt_*(APP)*_ or L-Apt.4–1c_5′hexynyl. As illustrated in Supplementary Figure S18, L-Apt.4–1c_5′hexynyl did not affect the *APP* mRNA levels, whereas L-Apt.4–1c-ASO15nt*_(APP)_* significantly reduced the *APP* mRNA levels, suggesting that L-Apt.4–1c_5′hexynyl inhibited *APP* translation mainly by stabilizing the rG4 structure, whereas L-Apt.4–1c-ASO15nt*_(APP)_* can also recruit RNase H to knock down *APP* mRNA in addition to inhibiting translation. It has been reported that RNase H1 exists in the cytosol of HeLa cells (52). To verify this, we performed RNase H1 subcellular distribution analysis by immunostaining using an RNase H1 antibody together with fluorescently labeled secondary antibody detected by confocal microscopy. Consistent with the fractionation experiments reported previously (52), our confocal microscopy results showed that RNase H1 was indeed present in the cytosol (Supplementary Figure S19). To examine the RNase H effect of our L-aptamer–ASO tool, we also conducted *in vitro* RNase H cleavage assay (Figure 5H). As expected, the cleavage result showed that the *APP* rG4 region alone did not induce RNase H cleavage (Figure 5I, Lane 2), whereas the *APP* rG4 region–L-Apt.4–1c-ASO15nt_*(APP)*_ complex was cleaved, generating a cleavage product that could be visualized on denaturing polyacrylamide gel (Figure 5I, Lane 4). The cleavage activity was both dosage- and time-dependent (Supplementary Figure S20). Overall, we demonstrated that L-aptamer–ASO conjugates can inhibit APP protein expression, and the inhibition is mediated by both translation suppression and RNase H-mediated mRNA knockdown.

### L-Apt.4-1c-ASO15nt*_(APP)_* can suppress *APP* rG4–DHX36 interactions and control DHX36-dependent *APP* rG4-mediated gene expression

**Figure 6.**
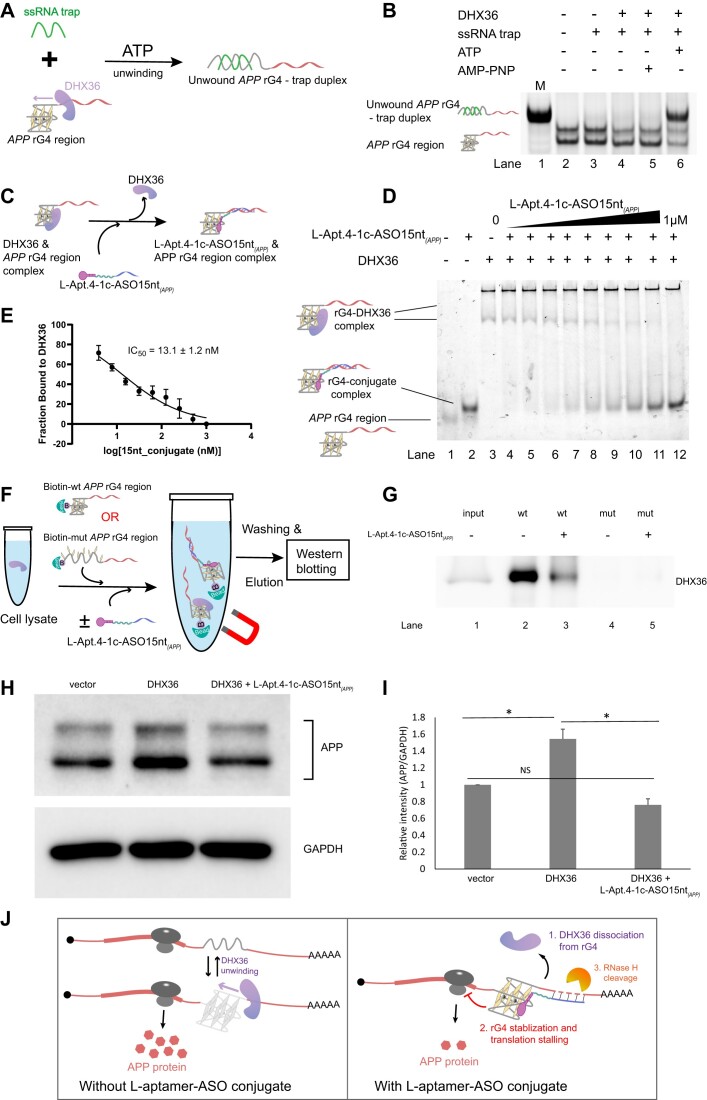
L-Apt.4–1c-ASO15nt_*(APP)*_ can dissociate *APP* rG4–DHX36 interactions, and control the DHX36-dependent *APP* rG4-mediated gene expression. (**A**) Schematic illustration of *APP* rG4 unwinding by DHX36 protein in an ATP-dependent manner. The ssRNA trap was designed to complementarily pair with the *APP* rG4 sequence. In the folded rG4 conformation, the ssRNA trap was unable to bind to *APP* rG4. Only when the rG4 was unwound by DHX36 did base paring occur between *APP* rG4 sequence and ssRNA trap. (**B**) DHX36 unwound *APP* rG4 in the presence of ATP, while not in the ATP non-hydrolysable analog, AMP-PNP. The ssRNA trap hybridized with the unwound FAM_*APP* rG4 region, showing unwound *APP* rG4–trap duplex shift bands. M: ssRNA trap was boiled together with FAM_*APP* rG4 region to form the duplex as a shift marker in the native polyacrylamide gel. (**C**) Schematic illustration of L-Apt.4–1c-ASO15nt_*(APP)*_ conjugate dissociating *APP* rG4–DHX36 protein interactions. **(D)** Inhibition assay showing that L-Apt.4–1c-ASO15nt_*(APP)*_ conjugate can effectively disrupt the interaction between HEX_*APP* rG4 wt region and DHX36 protein. (**E**) Inhibition curve obtained from (**D**). The IC_50_ value was determined to be 13.1 ± 1.2 nM. (**F**) Schematic illustration of RNA immunoprecipitation (RIP) assay. (**G**) L-Apt.4–1c-ASO15nt_(APP)_ conjugate can disrupt *APP* rG4–DHX36 interactions according to RIP assay (100 nM L-Apt.4–1c-ASO15nt_(*APP)*_). (**H**) DHX36 overexpression up-regulated APP expression, while L-Apt.4–1c-ASO15nt_*(APP)*_ rescued the effect of DHX36 according to western blotting. GAPDH was used as loading control. (**I**) Normalized APP expression results obtained from (**H**). (**J**) Schematic illustration of the mechanism by which L-Apt.4–1c-ASO15nt_*(APP)*_ regulates *APP* expression. Results were obtained from three replicates. Error bars represent the standard error of the mean. *P* values were calculated using two-tailed *t*-tests, with *P* < 0.05 considered to indicate significant differences.

Studies have previously reported that DHX36 is an important rG4-binding protein that can unwind the rG4 structure, thereby regulating gene expression and influencing disease development (53,54). However, whether DHX36 can bind and unwind *APP* rG4 remains unclear. To test this, we performed a binding assay between *APP* rG4 and DHX36 using EMSA, and strong gel shift bands were observed (*K*_d_ = 51.3 nM) when DHX36 protein was added, showing that DHX36 protein binds well to the *APP* rG4 region (Supplementary Figure S21). Next, we explored the unwinding ability of DHX36 on *APP* rG4. We designed an ssRNA trap oligonucleotide that is complementary to the unfolded rG4 sequence. When rG4 is unwound, the ssRNA trap hybridizes with the unfolded rG4 sequence and forms a partial duplex to prevent refolding of the rG4 structure, resulting in reduced mobility bands on a native polyacrylamide gel, by which the process of DHX36 unwinding can be measured (Figure 6A). Because DHX36 unwinding activity is reported to be ATP-dependent (55), we also tested its activity using the non-hydrolysable analog AMP-PNP as a negative control. To clearly show the shifted bands, a positive shift band (unwound *APP* rG4–trap duplex) as marker was produced by directly hybridizing the ssRNA trap and *APP* rG4 region sequence through boiling and annealing (Figure 6B, Lane 1). As shown in Figure 6B, in the presence of the ssRNA trap, DHX36 protein, and ATP, a shift band (unwound *APP* rG4–trap duplex) appeared (Figure 6B, Lane 6). In contrast, there were no shift bands observed when only DHX36 protein (Figure 6B, Lane 4) or DHX36 protein and AMP-PNP were present (Figure 6B, Lane 5). This suggests that DHX36 can unwind the *APP* rG4 structure *in vitro* in an ATP-dependent manner.

To extend the application of L-aptamer–ASO conjugates, we attempted to determine whether the conjugate can affect the interaction between the *APP* rG4 region and DHX36 protein *in vitro*. A competitive binding assay was thus performed. As shown schematically in Figure 6C, the *APP* rG4 region and DHX36 protein can form a complex, and the addition of L-Apt.4–1c-ASO15nt*_(APP)_* can disrupt the complex, thereby forming a new *APP* rG4 region–L-Apt.4–1c-ASO15nt*_(APP)_* complex. The EMSA result showed that L-Apt.4–1c-ASO15nt*_(APP)_* can efficiently dissociate the binding between *APP* rG4 region and DHX36 protein, judging from the decreasing amount of *APP* rG4 region–DHX36 complex and the increasing amount of *APP* rG4 region–L-Apt.4–1c-ASO15nt*_(APP)_* complex with increasing concentration of L-Apt.4–1c-ASO15nt*_(APP)_* (Figure 6D), with a calculated IC_50_ of 13.1 ± 1.2 nM (Figure 6E). We also performed *in vitro* dissociation assay of the *APP* rG4 region–DHX36 with L-Apt.4–1c_5′hexynyl only and with ASO15nt*_(APP)_*only as additional controls. We found that neither L-Apt.4–1c_5′hexynyl only nor ASO15nt*_(APP)_* could disrupt *APP* rG4–DHX36 interaction (Supplementary Figure S22). The structural domain of the DHX36 protein, the RHAU53 peptide, is responsible for binding to rG4s (56). To confirm whether L-Apt.4–1c-ASO15nt*_(APP)_* disrupted the binding of DHX36 and *APP* rG4 through competition with the RHAU53 domain, we examined the disruptive effect of the L-Apt.4–1c-ASO15nt*_(APP)_* on the binding of the RHAU53 peptide to the *APP* rG4 region. The EMSA result showed that L-Apt.4–1c-ASO15nt_*(APP)*_ could inhibit their binding efficiently (Supplementary Figure S23). Next, to establish whether the *APP* rG4 region can interact with the DHX36 in cells, we performed RIP assay by incubating the biotin-labeled *APP* rG4 region (Biotin-wt *APP* rG4 region) with cell lysate, using the Biotin-mut *APP* rG4 region as a negative control (Figure 6F). The RIP result showed that DHX36 protein bound to the wt *APP* rG4 region (Figure 6G, Lane 2), but not to the mut *APP* rG4 region (Figure 6G, Lane 4), indicating that the interaction between *APP* rG4 and DHX36 was rG4 structure-dependent. After additional L-Apt.4–1c-ASO15nt_*(APP)*_ treatment, the binding of DHX36 to the wt *APP* rG4 region decreased (Figure 6G, Lanes 2 and 3), suggesting that the L-Apt.4–1c-ASO15nt_*(APP)*_ conjugate can disrupt *APP* rG4–DHX36 interactions under cellular conditions. Having investigated the effect of L-Apt.4–1c-ASO15nt_*(APP)*_ on DHX36 *in vitro* and under cellular conditions, we wondered whether L-Apt.4–1c-ASO15nt_*(APP)*_ can also affect DHX36 function in cells. We overexpressed DHX36 by transfecting HeLa cells with DHX36-pcDNA3.1–3 × Flag-C plasmid, with pcDNA3.1–3 × Flag-C vector transfected as a negative control. Western blotting results showed that compared with the vector group, DHX36 overexpression elevated the APP protein levels through the unwinding of *APP* rG4. However, when cells were co-transfected with L-Apt.4–1c-ASO15nt_*(APP)*_ and DHX36 plasmid, the APP protein levels were reduced compared with the DHX36 overexpression group (Figure 6H and I), suggesting that L-Apt.4–1c-ASO15nt_*(APP)*_ can rescue the DHX36 overexpression effect and control DHX36-dependent *APP* rG4-mediated gene expression. Overall, we showed that L-Apt.4–1c-ASO15nt_*(APP)*_ can affect DHX36 function both *in vitro* and in cells. Based on the data gathered, we proposed a working model for L-Apt.4–1c-ASO15nt_*(APP)*_ in cells (Figure 6J). Under normal conditions, *APP* rG4 is dynamically regulated by rG4 binding proteins, such as DHX36. When treated with L-Apt.4–1c-ASO15nt_*(APP)*_, DHX36 is dissociated from *APP* rG4, protecting the rG4 structure from unwinding by DHX36. At the same time, the conjugate binding to rG4 also stabilizes the rG4 structure, together intensifying the inhibition of translation. In addition, the hybridized ASO DNA and RNA duplex recruits and triggers RNase H cleavage of the RNA transcript, leading to knockdown of the mRNA level. In addition, we performed dissociation assay on other rG4–DHX36 interactions (*TRF2*) using L-Apt.4–1c-ASO15nt_*(APP)*_, L-aptamer only and ASO only. Neither L-Apt.4–1c-ASO15nt_*(APP)*_, L-Apt.4–1c_5′hexynyl, nor ASO15nt_*(APP)*_ showed any observable dissociation ability for the *TRF2* rG4 region (Supplementary Figure S24), which may be due to the stronger binding of the *TRF2* rG4 region-DHX36 or the weaker binding of the *TRF2* rG4 region and L-Apt.4–1c. Taken together, the mechanisms mediated by the L-aptamer–ASO allow the precise control of gene expression.

## Discussion

Over the past years, rG4-binding ligands, peptides and antibodies have been developed. However, most of them can only discriminate G4 from non-G4 structures (39–41). Despite the good selectivity of G4-targeting L-aptamers (47–49), it remains challenging to recognize an individual rG4 of interest. In this proof-of-concept study, we propose an innovative approach of integrating an ASO complementary to the rG4 flanking region (for sequence recognition) and a general rG4 binder, L-Apt.4–1c (for structure recognition), to improve rG4-targeting specificity and affinity. We provide a general click-chemistry synthesis method of the L-aptamer–ASO conjugate, specifically copper-catalyzed azide–alkyne cycloaddition, between azide-modified ASO DNA and hexynyl-modified L-Apt.4–1c (Figure 1). Click chemistry is a powerful tool in modern synthetic chemistry for efficient and selective covalent bond formation under mild conditions (57). We designed three different lengths of ASO for binding evaluation and optimization, i.e. 10, 15 and 20 nt, and found that the 15- and 20-nt ASOs showed superior ability to that of the 10-nt ASO (Figure 2, S7 and S17). Considering the potential cost and cell penetration ability, we selected the 15-nt ASO for downstream experiments, i.e. L-Apt.4–1c-ASO15nt_*(APP)*_. Regarding the binding specificity and affinity, we found that L-Apt.4–1c-ASO15nt_*(APP)*_ specifically recognized the *APP* rG4 region in preference to the other rG4s, dG4s and non-G4s tested (Figure 3), and showed much weaker binding to the *Bcl2*, *TRF2*, and *MT3-MMP* rG4 regions (Supplementary Figures S13 and S14), which were previously reported to bind L-Apt.4–1c (47). The binding affinity of L-Apt.4–1c-ASO15nt*_(APP)_* was stronger than that of L-Apt.4–1c alone, with *K*_d_ values of 0.4 and 60.4 nM, respectively. These results confirm that our bi-functional recognition strategy using two different recognition modules can not only achieve precise targeting of the desired rG4 region of interest but can also increase the binding affinity.

It is worth noting that several groups have attempted to utilize G4 flanking sequence complementary oligonucleotides to target individual G4s. Chen *et al.* developed a G4-triggered fluorogenic hybridization probe to uniquely visualize *NRAS* rG4 in cells (58). Dai *et al.* developed Module Assembled Multifunctional Probes Assay, which utilized a carboxypyridostatin ligand-oligonucleotide to visualize single rG4 in cells (59). In the present work, we also comprehensively applied our L-aptamer–ASO conjugate to intracellular rG4 imaging and found that it could efficiently distinguish *APP* rG4 RNA from other rG4 RNAs (Figure 4). To the best of our knowledge, this is the first time that an L-aptamer–ASO conjugate has been used to image an individual rG4 of interest in cells, providing one more rG4-specific probe for imaging purposes. Tassinari *et al.* constructed a naphthalene diimide (NDI)–peptide nucleic acid (PNA) conjugate to specifically recognize the dG4 region within the human immunodeficiency virus type 1 (HIV-1) long terminal repeat (LTR) (60). Berner *et al.* reported selective targeting of *c-MYC* Pu24T dG4 by a quinazoline–pyrimidine ligand–oligonucleotide conjugate (GL-O) (61). Compared with the above studies, ours has several advantages. (1) The modification ability of the conjugate is more diverse due to its composition of multiple nucleotides. (2) The materials can be easily obtained and the method is especially suitable for biochemists and biologists who do not have the apparatus and experience to perform the chemical synthesis. (3) The L-aptamer-conjugate binds much more strongly to the target G4. The *K*_d_ between our L-Apt.4–1c-ASO15nt*_(APP)_* and the *APP* rG4 region was 0.4 nM, whereas that of GL-O15 to *c-MYC* Pu24T dG4 was 12 nM (61) [the *K*_d_ between NDI–PNA and HIV-1 LTR dG4 was not measured (60)].

In terms of application, most of the above studies performed *in vitro* experiments (60,61). Tassinari *et al.* observed the uptake of the NDI–PNA conjugate directly into cells by confocal microscopy (60), but no further cellular function was validated. Here, we broadened the cellular application possibilities using our L-aptamer–ASO tool, and for the first time applied the new tool to control endogenous *APP* gene expression by targeting the rG4 region (Figure 5 and Supplementary Figure S17). Importantly, we also delved into the mechanism by which the conjugate represses gene expression. Consistent with other previously reported L-aptamers (48,51,62), the L-Apt.4–1c-ASO15nt*_(APP)_* conjugate can inhibit translation of the targeted gene. Additionally, the L-Apt.4–1c-ASO15nt*_(APP)_* conjugate showed an mRNA knockdown effect by RNase H cleavage (Figure 5 and Supplementary Figures S18 and S20). RNase H can recognize DNA:RNA heteroduplexes and cleave the RNA region. This finding highlights the combined effect of the two recognition modules, in which the L-aptamer binds and stabilizes the rG4 structure, and the ASO enables RNase H-mediated mRNA degradation, underscoring the importance of chemical design in realizing a novel mechanism of action. However, ASO alone failed to suppress gene expression in cells in both the reporter gene assay (Supplementary Figure S16) and endogenous *APP* (Supplementary Figure S25), probably due to ASO being degraded within the cells. Furthermore, we applied L-Apt.4–1c-ASO15nt_*(APP)*_ conjugate to affect the rG4-binding protein DHX36 *in vitro* and intracellularly. DHX36 can unwind the rG4 secondary structure (55), and we demonstrated for the first time that DHX36 can unfold *APP* rG4 (Figure 6A and B). We also showed that L-Apt.4–1c-ASO15nt*_(APP)_* dissociated the DHX36–*APP* rG4 interaction (Figure 6C–I), revealing an additional mechanism of the regulation of gene expression through rG4 structure stabilization. This observation not only reveals the functional role of rG4-binding proteins, but also highlights the ability of the L-aptamer–ASO conjugate to modulate these interactions, opening avenues for further tool development and G4-mediated gene control in different RNA transcripts.

## Conclusion

In this work, we have rationally designed a robust approach to conjugate an L-aptamer and a D-ASO using click chemistry, and developed a novel G4-targeting L-aptamer–ASO conjugate that can specifically recognize an individual rG4 region of interest with sub-nanomolar binding affinity. Using *APP* rG4 as the example, we applied the L-aptamer–ASO conjugate (i.e. L-Apt.4–1c-ASO15nt_*(APP)*_) to control DHX36-dependent *APP* gene expression via targeting the *APP* 3′ UTR rG4. Furthermore, we deciphered the working mechanism of the L-aptamer–ASO conjugate in gene control, which involves impairing the unwinding of the rG4 structure by DHX36 while also inducing both translational repression and RNase H-mediated mRNA knockdown. Notably, our new conjugate system is modular and can be easily adapted to target other rG4s or nucleic acid secondary structures of interest by simply replacing the ASO sequence and/or L-aptamer. Considering the emerging roles of G4s in biological processes and diseases, we think that this innovative toolset can be further applied to deepen our understanding of G4-mediated gene expression and provide valuable insights into potential treatment of G4-associated diseases.

## Supplementary Material

gkae1034_Supplemental_File

## Data Availability

All the data generated during the experiments are available from the author upon reasonable request.
