# Supplementary material for: Selective recognition of RNA G-quadruplex in vitro and in cells by L-aptamer–D-oligonucleotide conjugate
Source: Nucleic Acids Res. 2024 Nov 18;52(22):13544–60. doi: 10.1093/nar/gkae1034 (PMC11662670; doi:10.1093/nar/gkae1034)
Supplement: gkae1034_Supplemental_File [file gkae1034_supplemental_file.pdf]

Supplementary Information

Selective recognition of RNA G-quadruplex *in vitro* and in cells by L-aptamer – D-oligonucleotide conjugate

Haizhou Zhao[1], Hill Lam Lau[1], Kun Zhang[1], Chun Kit Kwok[1,2,*]

[1] Department of Chemistry and State Key Laboratory of Marine Pollution, City University of Hong Kong, Kowloon Tong, Hong Kong SAR, China

[2] Shenzhen Research Institute of City University of Hong Kong, Shenzhen, China

*To whom correspondence should be addressed. Tel: +852 3442 6858; Fax: +852 3442 0522; Twitter: @kitkwok6; Email: ckkwok42@cityu.edu.hk

Table of contents

Table S1. Oligonucleotides used in this study.

Table S2. Proteins and peptides used in this study.

Figure S1. Mass spectrometry of L-Apt.4-1c_5'Hexynyl.

Figure S2. Mass spectrometry of ASO 10nt_DNA_3'Azide.

Figure S3. Mass spectrometry of ASO 15nt_DNA_3'Azide.

Figure S4. Mass spectrometry of ASO 20nt_DNA_3'Azide.

Figure S5. Mass spectrometry of ASO 15nt_DNA-5'FAM-3'Azide.

Figure S6. L-Apt.4-1c binds strongly to *APP* D-rG4 motif with nanomolar affinity.

Figure S7. L-Apt.4-1c_5'Hexynyl binds with nanomolar affinity to *APP* D-rG4 in the presence or absence of flanking sequence.

Figure S8. Synthesis of L-Apt.4-1c-ASO conjugate by click reaction.

Figure S9. MALDI TOF mass spectrometry of L-Apt.4-1c-ASO10nt$_{(APP)}$ conjugate.

Figure S10. MALDI TOF mass spectrometry of L-Apt.4-1c-ASO15nt$_{(APP)}$ conjugate.

Figure S11. MALDI TOF mass spectrometry of L-Apt.4-1c-ASO20nt$_{(APP)}$ conjugate.

Figure S12. L-Apt.4-1c-ASO conjugates exhibit a markedly reduced binding capacity toward *APP* rG4 mut region analyzed by EMSA.

Figure S13. L-Apt.4-1c-ASO conjugate shows no enhanced binding to *Bcl2* rG4 region, *TRF2* rG4 region and *MT3-MMP* rG4 region analyzed by EMSA assay.

Figure S14. L-Apt.4-1c-ASO conjugate shows enhanced binding to *APP* D-rG4 region analyzed by MST assay.

Figure S15. Synthesis of FAM_L-Apt.4-1c-ASO15nt$_{(APP)}$ conjugate for the specific recognition of *APP* rG4 region in cell imaging assay.

Figure S16. Dual luciferase reporter gene assay showed that L-Apt.4-1c-ASO15nt$_{(APP)}$ and L-Apt.4-1c_5'Hexynyl can negatively regulated gene expression via targeting *APP* rG4 structure.

Figure S17. Inhibitory effect of L-Apt.4-1c-ASO10nt$_{(APP)}$, L-Apt.4-1c-ASO15nt$_{(APP)}$, and L-Apt.4-1c-ASO20nt$_{(APP)}$ on endogenous APP protein level in Hela cells detected by western blotting.

Figure S18. L-Apt.4-1c-ASO15nt$_{(APP)}$ conjugate knock down endogenous *APP* mRNA level in cells analyzed by RT-qPCR.

Figure S19. RNase H1 subcellular distribution analyzed by confocal microscopy.

Figure S20. The cleavage of the conjugate-*APP* rG4 region complex by RNase H is contingent on the dosage levels and duration of exposure.

Figure S21. DHX36 protein binds well to *APP* rG4 region analyzed by EMSA.

Figure S22. Inhibition assay shows that neither L-Apt.4-1c_5'Hexynyl nor ASO15nt$_{(APP)}$ can disrupt the interaction between *APP* rG4 region and DHX36 protein.

Figure S23. L-Apt.4-1c-ASO15nt$_{(APP)}$ conjugate can dissociate *APP* rG4 – RHAU53 interaction analyzed by EMSA.

Figure S24. Inhibition assay shows that L-Apt.4-1c-ASO15nt$_{(APP)}$, L-Apt.4-1c_5'Hexynyl, and ASO15nt$_{(APP)}$ cannot disrupt the interaction between *TRF2* rG4 region and DHX36 protein.

Figure S25. ASO itself cannot inhibit endogenous *APP* expression analyzed by western blotting.

Table S1. Oligonucleotides used in this study.

Name	Sequences (5’-3’)
L-Apt.4-1c_5’Hexynyl	L-Hexynyl-GCCCUAAAGGUGGUGGUGGGAGGGC
ASO 10nt_DNA_3’Azide	CAGCAGAGCA-Azide
ASO 15nt_DNA_3’Azide	AAGACCAGCAGAGCA-Azide
ASO 20nt_ DNA_3’Azide	AATTGAAGACCAGCAGAGCA-Azide
ASO 15nt_DNA-5’FAM-3’Azide	FAM-AAGACCAGCAGAGCA-Azide
FAM_*TRF2* rG4 region	FAM-GGGAGGGCGGGGAGGGCGCGCGGCGAUCGGACACGA
FAM_*MT3-MMP* rG4 region	FAM-GAGGGAGGGAGGGAGAGGGAGAGAGGGAGAAAACGAAGGG
FAM_*Bcl2* rG4 region	FAM-GGGGGCCGUGGGGUGGGAGCUGGGGCGAGAGGUGCCGUUGGCCCC
FAM_*APP* rG4 wt region	FAM-GGGGCGGGUGGGGAGGGGUGCUCUGCUGGUCUUCAAUU
FAM_*APP* rG4 wt motif	FAM-CGGGGCGGGUGGGGAGGGGU
FAM_*APP* rG4 mut motif	FAM-CGAAGCGAGUGAAGAGAAGU
HEX_*APP* rG4 wt region	HEX-GGGGCGGGUGGGGAGGGGUGCUCUGCUGGUCUUCAAUU
HEX_*APP* rG4 mut region	HEX-GAAGCGAGUGAAGAGAAGUGCUCUGCUGGUCUUCAAUU
FAM_*c-Kit 1* dG4 region	FAM-GGAGGGCGCTGGGAGGAGGGGCTGCTGCTCGCCGCTCGCG
FAM_*hTERC* dG4 region	FAM-GGGTTGCGGAGGGTGGGCCTGGGAGGGGTGGTGGCCA
FAM_*hTELO* dG4 region	FAM-TTAGGGTTAGGGTTAGGGTTAGGGTTAGAGTTAGAGTTAGAGTT
FAM_*Bcl2* Mid dG4 region	FAM-GGGCGCGGGAGGAAGGGGGCGGGAGCGGGGCTGTGGTGCCTGT
FAM_*VEGF* dG4 region	FAM-GGGGCGGGCCGGGGGCGGGGTCCCGGCGGGGCGGAGCCAT
FAM_*Bcl2* dG4 region	FAM-GGGGGCCGTGGGGTGGGAGCTGGGGCGAGAGGTGCCGTTGGCCCC
FAM_*MT3-MMP* dG4 region	FAM-GAGGGAGGGAGGGAGAGGGAGAGAGGGAGAAAACGAAGGG
FAM_*TRF2* dG4 region	FAM-GGGAGGGCGGGGAGGGCGCGCGGCGATCGGACACGA
poly rA	FAM-AAAAAAAAAAAAAAAAAA
poly rC	FAM-CCCCCCCCCCCCCCCCCC
poly rU	FAM-UUUUUUUUUUUUUUUUUU

DNA hairpin	FAM-CAGTACAGATCTGTACTG
RNA hairpin	FAM-CAGUACAGAUCUGUACUG
ssRNA trap	UCCCCACCCGCCCCG
Biotin-wt *APP* rG4 region	Biotin-GGGGCGGGUGGGGAGGGGUGCUCUGCUGGUCUU
Biotin-mut *APP* rG4 region	Biotin-GAAGCGAGUGAAGAGAAGUAAUAUACUAAUAUU
T7-imaging-forward DNA strand	TAATACGACTCACTATAG
APP wt-imaging-reverse DNA strand	TACAATCATCCTGCAGAAAATTGTTTTGGAGAATTCTTGGTAATTGAAGACCAGCAGAGCACCCCTCCCCACCCGCCCCGTAAAAGTGCTTACAATGAACAGGGATTCTTTTCTTTATCAAAGACTATAGTGAGTCGTATTA
APP rG4 region deleted-imaging-reverse DNA strand	TACAATCATCCTGCAGAAAATTGTTTTGGAGAATTCTTGGTAATTGGTAAAAGTGCTTACAATGAACAGGGATTCTTTTCTTTATCAAAGACTATAGTGAGTCGTATTA
TRF2-imaging-reverse DNA strand	TGCCCGCCCGCTGCCGTCGCTACTCCCGCCTCCTCCCGCCATCGTGTCCGATCGCCGCGCCCTCCCCGCCCTCCCGGCCGGGCCGCTTCCTCGGCTGTGACGCCGCTGGGTCACGCACGACTATAGTGAGTCGTATTA
MT3-MMP-imaging-reverse DNA strand	TAAGATCATAGTGAACTGTGCTTCAATGGATGGACGAGCTCCCCTTCGTTTTCTCCCTCTCTCCCTCTCCCTCCCTCCCTCGTTTCCTTTCAAAAAAAAGTCCTCCGGGTGGGTAAGGAGCCTGCTATAGTGAGTCGTATTA
APP Cy3-probe	Cy3-TACAATCATCCTGCAGAAAATTGTT
TRF2 Cy3-probe	Cy3-TGCCCGCCCGCTGCCGTCGCTACTC
MT3-MMP Cy3-probe	Cy3-TAAGATCATAGTGAACTGTGCTTCA
GAPDH-forward primer	GGAGCGAGATCCCTCCAAAAT
GAPDH-reverse primer	GGCTGTTGTCATACTTCTCATGG
18S-forward primer	CGGCGACGACCCATTCGAAC
18S-reverse primer	GAATCGAACCCTGATTCCCCGTC
APP-forward primer	GTGCTCTGCTGGTCTTCAAT
APP-reverse primer	GAAAAGTCTTGCCCGGGGTT
APP'-forward primer	GCCCTGCGGAATTGACAAG
APP'-reverse primer	CCATCTGCATAGTCTGTGTCTG
wt *APP* rG4 region-reporter insertion	TTGGGTCTTTGATAAAGAAAAGAATCCCTGTTCATTGTAAGCACTTTTACGGGGCGGGTGGGGAGGGGTGCTCTGCTGGTCTTCAATTACCAAGAATTCTCCAAAACAATTTTCTGCAGGATGATTGTA
mut *APP* rG4 region-reporter insertion	TTGGGTCTTTGATAAAGAAAAGAATCCCTGTTCATTGTAAGCACTTTTACGAAGCGAGTGAAGAGAAGTAATATACTAATATTCAATTACCAAGAATTCTCCAAAACAATTTTCTGCAGGATGATTGTA
DHX36 ORF	ATGAGTTATGACTACCATCAGAACTGGGGCCGTGATGGGGGTCCCCGCAGCTCCGGTGGGGGCTATGGAGGGGGGCCAGC

	AGGGGGTCATGGAGGTAACCGAGGCTCCGGAGGAGGCGGC<br>GGCGGCGGAGGGGGTGGTCGAGGCGGCAGGGGCCGGCATC<br>CCGGGCACCTGAAAGGCCGCGAAATCGGCATGTGGTACGC<br>GAAAAAACAGGGGCAGAAGAACAAGGAAGCGGAGAGGCAA<br>GAGAGAGCTGTAGTACACATGGATGAACGACGAGAAGAAC<br>AAATTGTACAGTTACTGAATTCTGTTCAAGCGAAGAATGA<br>TAAAGAGTCAGAAGCACAGATATCCTGGTTTGCTCCTGAG<br>GATCATGGATACGGTACTGAAGTTTCTACTAAGAACACAC<br>CATGCTCAGAGAACAAACTTGACATCCAGGAAAAGAAGTT<br>GATAAATCAAGAAAAAAAAATGTTTAGAATCAGGAACAGA<br>TCATATATTGACCGAGATTCTGAGTATCTCTTGCAAGAAA<br>ATGAACCAGATGGAACTTTAGACCAAAAATTATTGGAAGA<br>TTTACAAAAGAAAAAAAATGACCTTCGGTATATTGAAATG<br>CAGCATTTCAGAGAAAAGCTGCCTTCGTATGGAATGCAAA<br>AGGAATTGGTAAATTTAATTGATAACCATCAGGTAACAGT<br>AATAAGTGGTGAAACTGGTTGTGGCAAAACCACTCAAGTT<br>ACTCAGTTCATTTTGGATAACTACATTGAAAGAGGAAAAG<br>GATCTGCTTGCAGAATAGTTTGTACTCAGCCAAGAAGAAT<br>TAGTGCCATTTCAGTTGCGGAAAGAGTAGCTGCAGAAAGG<br>GCAGAATCTTGTGGCAGTGGTAATAGTACTGGATATCAAA<br>TTCGTCTCCAGAGTCGGTTGCCAAGGAAACAGGGTTCTAT<br>CTTATACTGTACAACAGGAATCATCCTTCAGTGGCTCCAG<br>TCAGACCCGTATTTGTCCAGTGTTAGTCATATCGTACTTG<br>ATGAAATCCATGAAAGAAATCTGCAGTCAGATGTTTTAAT<br>GACTGTTGTTAAAGACCTTCTCAATTTTCGATCTGACTTG<br>AAAGTAATATTGATGAGTGCAACATTGAATGCAGAAAAGT<br>TTTCAGAATATTTTGGTAACTGTCCAATGATACATATACC<br>TGGTTTTACCTTTCCGGTTGTGGAATATCTTTTGGAAGAT<br>GTAATTGAAAAAATAAGGTATGTTCCAGAACAAAAAGAAC<br>ACAGATCCCAGTTTAAGAGGGGTTTCATGCAAGGGCATGT<br>AAATAGACAAGAAAAAGAAGAAAAAGAAGCAATATATAAA<br>GAACGTTGGCCAGATTATGTAAGGGAACTGCGAAGAAGGT<br>ATTCTGCAAGTACTGTAGATGTTATAGAAATGATGGAGGA<br>TGATAAAGTTGATCTGAATTTGATTGTTGCCCTCATCCGA<br>TACATTGTTTTGGAAGAAGAGGATGGTGCGATACTGGTCT<br>TTCTGCCAGGCTGGGACAATATCAGCACTTTACATGATCT<br>CTTGATGTCACAAGTAATGTTTAAATCAGTTAACCAGACA<br>CAGGTGTTTAAAAGAACCCCTCCTGGTGTTCGGAAAATAG<br>TAATTGCTACCAACATTGCGGAGACTAGCATTACCATAGA<br>TGATGTCGTTTATGTGATAGATGGAGGAAAAATAAAAGAG<br>ACACATTTTGATACTCAGAACAATATCAGTACAATGTCCG<br>CTGAGTGGGTTAGTAAAGCTAATGCCAAACAGAGAAAAGG<br>TCGAGCTGGAAGAGTTCAACCTGGTCATTGCTATCATCTG<br>TATAATGGTCTTAGAGCAAGTCTTCTAGATGACTATCAAC

	TGCCAGAAATTTTGAGAACTCCTTTGGAAGAACTTTGTTT<br>ACAAATAAAGATTTTAAGGCTAGGTGGAATTGCTTATTTT<br>CTGAGTAGATTAATGGACCCACCATCAAATGAGGCAGTGT<br>TACTCTCCATAAGACACCTGATGGAGCTGAACGCTTTGGA<br>TAAACAAGAAGAATTGACACCTCTTGGAGTCCACTTGGCA<br>CGATTACCCGTTGAGCCACATATTGGAAAAATGATTCTTT<br>TTGGAGCACTGTTCTGCTGCTTAGACCCAGTACTCACTAT<br>TGCTGCTAGTCTCAGTTTCAAAGATCCATTTGTCATTCCA<br>CTGGGAAAAGAAAAGATTGCAGATGCAAGAAGAAAGGAAT<br>TGGCAAAGGATACTAGAAGTGATCACTTAACAGTTGTGAA<br>TGCGTTTGAGGGCTGGGAAGAGGCTAGGCGACGTGGTTTC<br>AGATACGAAAAGGACTATTGCTGGGAATATTTTCTGTCTT<br>CAAACACACTGCAGATGCTGCATAACATGAAAGGACAGTT<br>TGCTGAGCATCTTCTTGGAGCTGGATTTGTAAGCAGTAGA<br>AATCCTAAAGATCCAGAATCTAATATAAATTCAGATAATG<br>AGAAGATAATTAAAGCTGTCATCTGTGCTGGTTTATATCC<br>CAAAGTTGCTAAAATTCGACTAAATTTGGGTAAAAAAAGA<br>AAAATGGTAAAAGTTTACACAAAAACCGATGGCCTGGTTG<br>CTGTTCATCCTAAATCTGTTAATGTGGAGCAAACAGACTT<br>TCACTACAACTGGCTTATCTATCACCTAAAGATGAGAACA<br>AGCAGTATATACTTGTATGACTGCACAGAGGTTTCCCCAT<br>ACTGTCTCTTGTTTTTTGGAGGTGACATTTCCATCCAGAA<br>GGATAACGATCAGGAAACTATTGCTGTAGATGAGTGGATT<br>GTATTTCAGTCTCCAGCAAGAATTGCCCATCTTGTTAAGG<br>AATTAAGAAAGGAACTAGATATTCTTCTGCAAGAGAAGAT<br>TGAAAGTCCTCATCCTGTAGACTGGAATGACACTAAATCC<br>AGAGACTGTGCAGTACTGTCAGCTATTATAGACTTGATCA<br>AAACACAGGAAAAGGCAACTCCCAGGAACTTTCCGCCACG<br>ATTCCAGGATGGATATTACAGC

Yellow highlighted regions are T7 promoter. Mutation was underlined and in red color.

Table S2. Proteins and peptides used in this study.

Name	Sequence
DHX36 protein	MSYDYHQNWGRDGGPRSSGGGYGGGPAGGHGGNRGSGGGGGGGGGR<br>GGRGRHPGHLKGREIGMWYAKKQGQKNKEAERQERAVVHMDERREEQ<br>IVQLLNSVQAKNDKESEAQISWFAPEDHGYGTEVSTKNTPCSENKLD<br>IQEKKLINQEKKMFRIRNRSYIDRDSEYLLQENEPDGTLDQKLLEDL<br>QKKKNDLRYIEMQHFREKLPSYGMQKELVNLIDNHQVTVISGETGCG<br>KTTQVTQFILDNYIERGKGSACRIVCTQPRRISAISVAERVAAERAE<br>SCGSGNSTGYQIRLQSRLPRKQGSILYCTTGIILQWLQSDPYLSSVS<br>HIVLDEIHERNLQSDVLMTVVKDLLNFRSDLKVILMSATLNAEKFSE<br>YFGNCPMIHIPGFTFPVVEYLLEDVIEKIRYVPEQKEHRCQFKRGFM<br>QGHVNRQEKEEKEAIYKERWPDYVRELRRRYSASTVDVIEMMEDDKV<br>DLNLIVALIRYIVLEEEDGAILVFLPGWDNISTLHDLLMSQVMFKSD<br>KFLIIPLHSLMPTVNQTQVFKRTPPGVRKIVIATNIAETSITIDDVV<br>YVIDGGKIKETHFDTQNNISTMSAEWVSKANAKQRKGRAGRVQPGHC<br>YHLYNGLRASLLDDYQLPEILRTPLEELCLQIKILRLGGIAYFLSRL<br>MDPPSNEAVLLSIRHLMELNALDKQEELTPLGVHLARLPVEPHIGKM<br>ILFGALFCCLDPVLTIAASLSFKDPFVIPLGKEKIADARRKELAKDT<br>RSDHLTVVNAFEGWEEARRRGFRYEKDYCWEYFLSSNTLQMLHNMKG<br>QFAEHLLGAGFVSSRNPKDPESNINSDNEKIIKAVICAGLYPKVAKI<br>RLNLGKKRKMVKVYTKTDGLVAVHPKSVNVEQTDFHYNWLIYHLKMR<br>TSSIYLYDCTEVSPYCLLFFGGDISIQKDNDQETIAVDEWIVFQSPA<br>RIAHLVKELRKELDILLQEKIESPHPVDWNDTKSRDCAVLSAIIDLI<br>KTQEKATPRNFPPRFQDGYYSTRTRPLEQKLISEEDLAANDILDYKD<br>DDDKV
RHAU53 peptide	HPGHLKGREIGMWYAKKQGQKNKEAERQERAVVHMDERREEQIVQLL<br>NSVQAK

Green highlighted: C-Myc / DDK tag

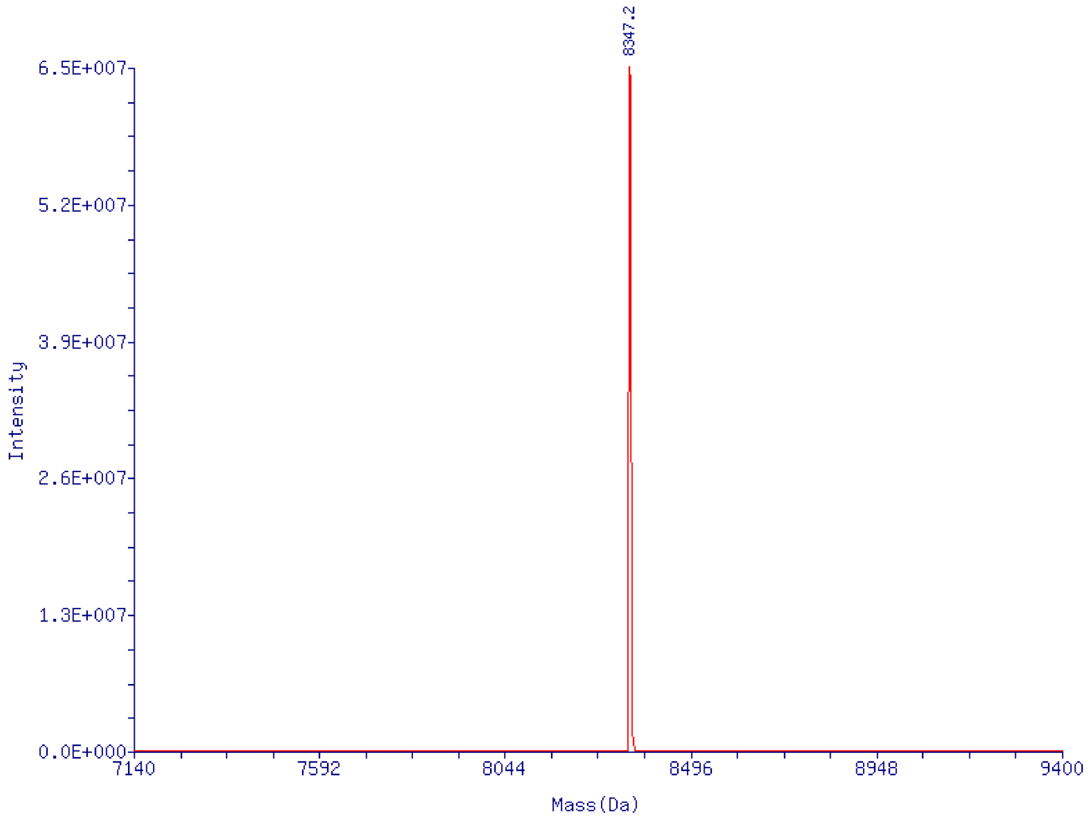

Figure S1. Mass spectrometry of L-Apt.4-1c_5'Hexynyl. Mass calculated: 8348.2, mass found: 8347.2. This suggests that L-Apt.4-1c_5'Hexynyl was synthesized and purified correctly.

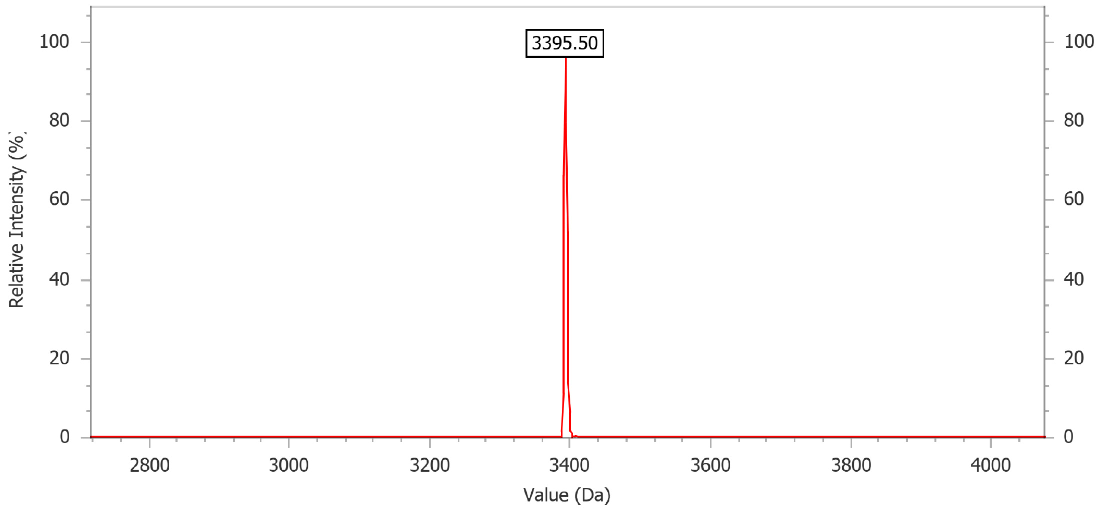

Figure S2. Mass spectrometry of ASO 10nt_DNA_3'Azide. Mass calculated: 3396.4, mass found: 3395.5. This suggests that ASO 10nt_DNA_3'Azide was synthesized and purified correctly.

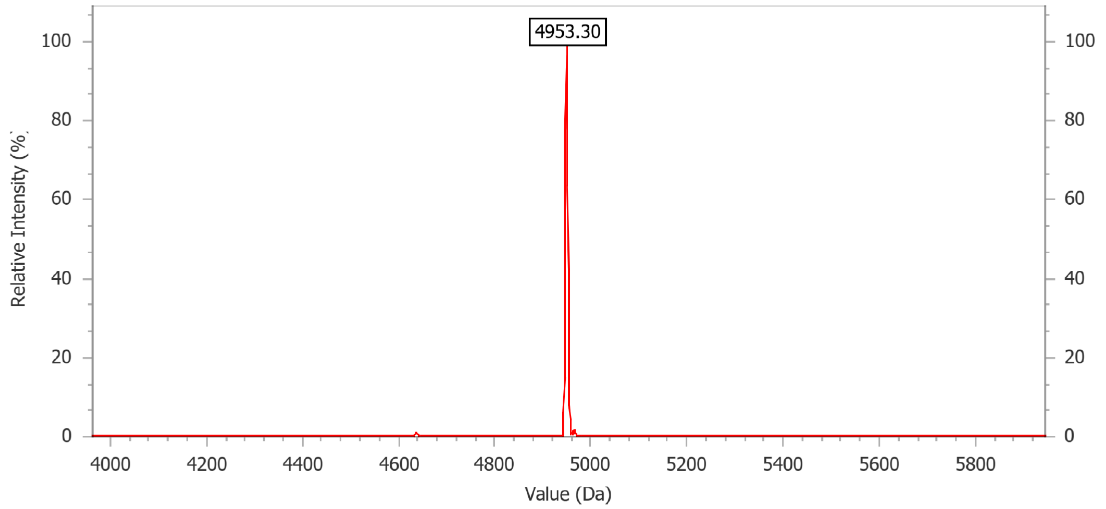

Figure S3. Mass spectrometry of ASO 15nt_DNA_3'Azide. Mass calculated: 4954.4, mass found: 4953.3. This suggests that ASO 15nt_DNA_3'Azide was synthesized and purified correctly.

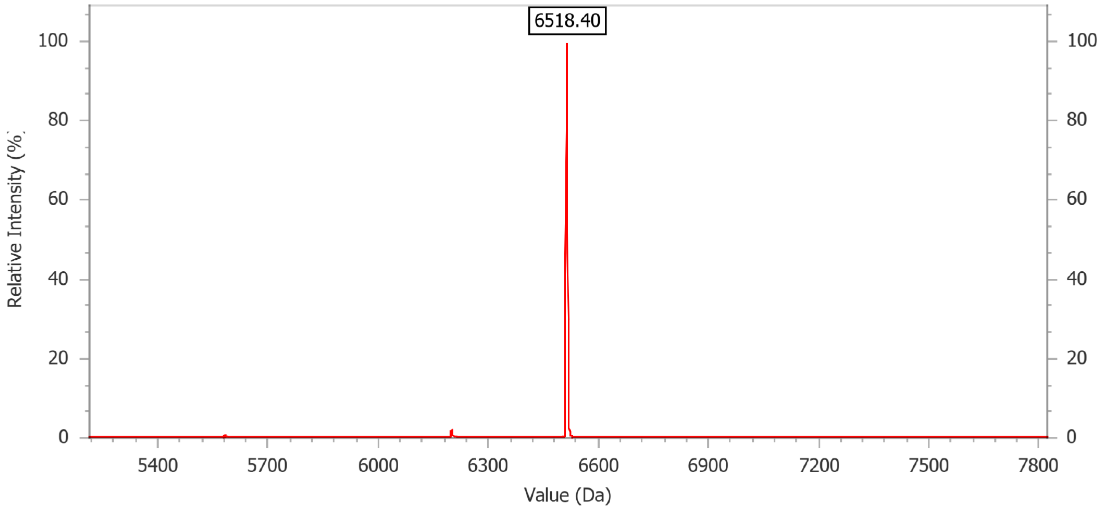

Figure S4. Mass spectrometry of ASO 20nt_DNA_3'Azide. Mass calculated: 6518.4, mass found: 6518.4. This suggests that ASO 20nt_DNA_3'Azide was synthesized and purified correctly.

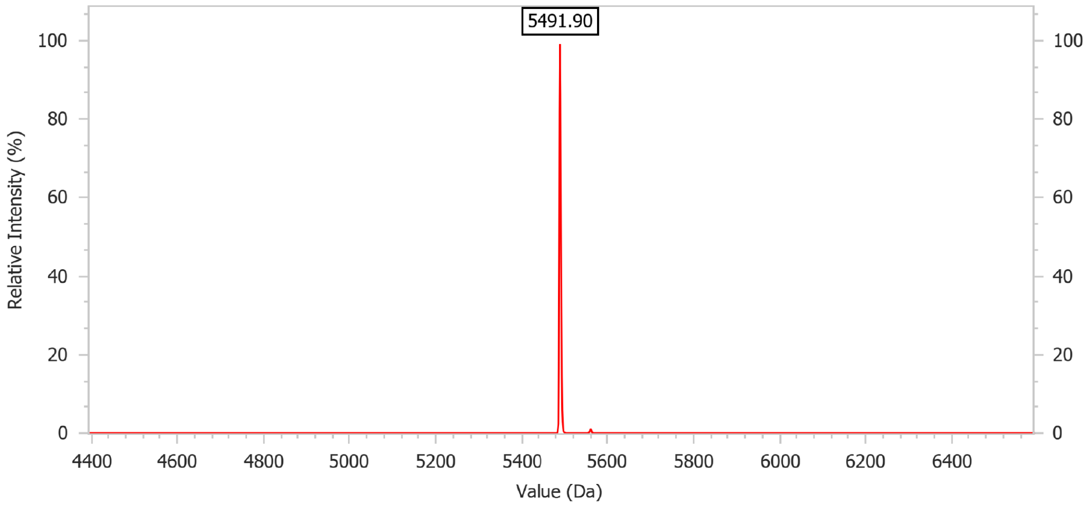

Figure S5. Mass spectrometry of ASO 15nt_DNA-5'FAM-3'Azide. Mass calculated: 5491.8, mass found: 5491.9. This suggests that ASO 15nt_DNA-5'FAM-3'Azide was synthesized and purified correctly.

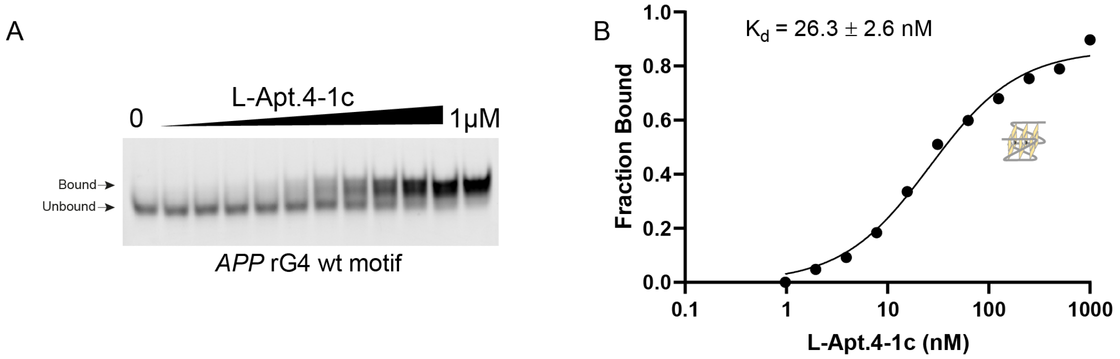

Figure S6. L-Apt.4-1c binds strongly to *APP* D-rG4 motif with nanomolar affinity. (A) The binding test between L-Apt.4-1c and the FAM_*APP* rG4 wildtype (wt) using EMSA. With increasing L-Apt.4-1c concentration, the intensity of the upper band (bound) becomes more intense, which indicated the interaction between *APP* rG4 target and L-Apt.4-1c. (B) Binding curve that displays the result from (A). K_d is calculated to be 26.3 ± 2.6 nM. *APP* rG4 wt motif used is 10 nM.

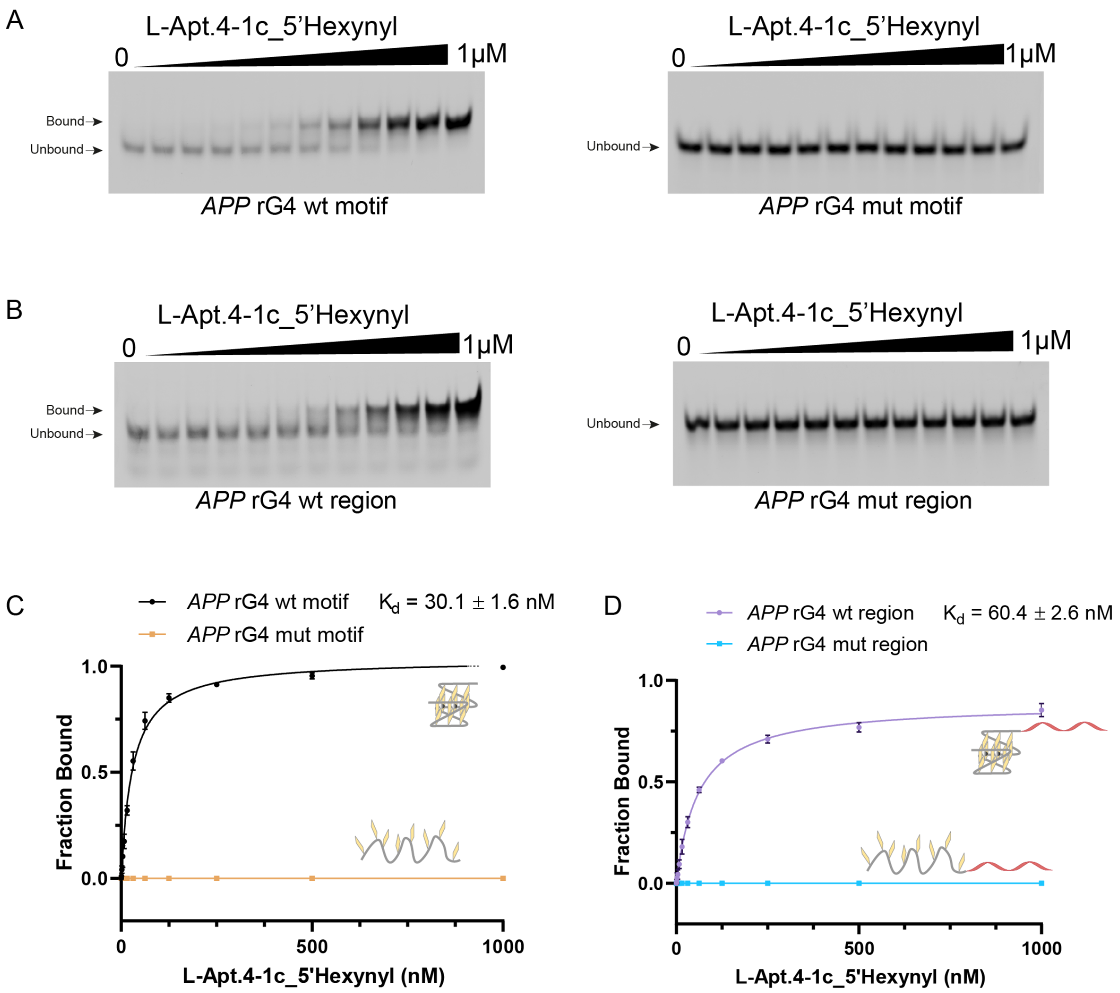

Figure S7. L-Apt.4-1c_5'Hexynyl binds with nanomolar affinity to *APP* D-rG4 in the presence or absence of flanking sequence. (A) Binding between L-Apt.4-1c_5'Hexynyl with FAM_*APP* rG4 wt or mutant (mut) motif analyzed by EMSA. Strong binding is observed for the *APP* rG4 wt motif, whereas not observed for the *APP* rG4 mut motif. (B) The binding association between L-Apt.4-1c_5'Hexynyl and HEX_*APP* rG4 wt or mut region analyzed by EMSA. Strong binding is observed for the *APP* rG4 wt region, but not for the *APP* rG4 mut region. (C) Binding curve of *APP* rG4 wt or mut motif against L-Apt.4-1c_5'Hexynyl from the EMSA results in (A). The K_d for *APP* rG4 wt motif is determined to be 30.1 ± 1.6 nM. No K_d can be obtained for the *APP* rG4 mut motif. (D) Binding curve of *APP* rG4 wt or mut region against L-Apt.4-1c_5'Hexynyl from the EMSA results in (B). The K_d for *APP* rG4 wt region is determined to be 60.4 ± 2.6 nM. No K_d can be obtained for the *APP* rG4 mut region. The presence of flanking sequence on *APP* rG4 only slightly weaken the L-Apt.4-1c_5'Hexynyl binding affinity. *APP* rG4 wt or mut motif / region used is 10 nM.

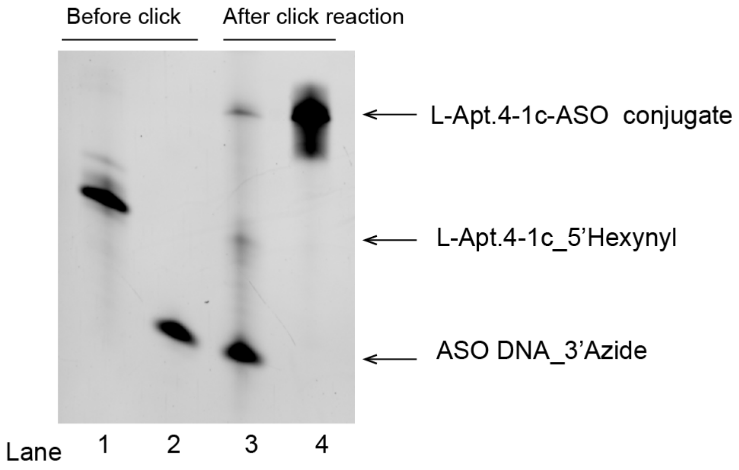

Figure S8. Synthesis of L-Apt.4-1c-ASO conjugate by click reaction. Lane 1: L-Apt.4-1c_5'Hexynyl, Lane 2: ASO DNA-3'Azide, Lane 3: ASO DNA-3'Azide reacted with L-Apt.4-1c_5'Hexynyl by click reaction to generate L-Apt.4-1c-ASO conjugate. Lane 4: The gel and column purified L-Apt.4-1c-ASO conjugate. The gel is stained by SYBR Gold.

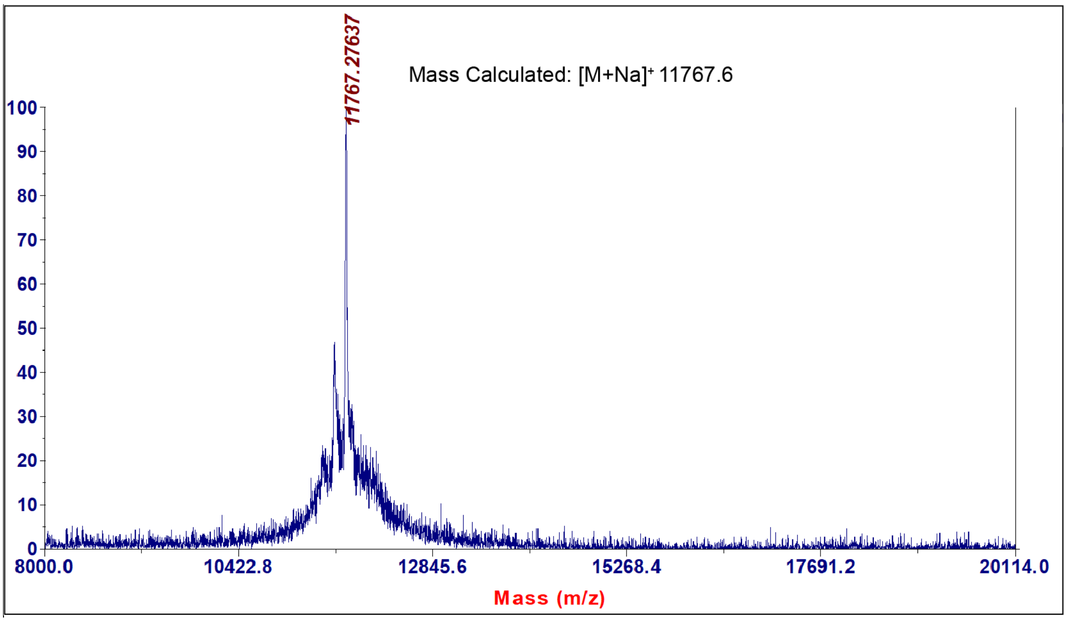

Figure S9. MALDI TOF mass spectrometry of L-Apt.4-1c-ASO10nt$_{(APP)}$ conjugate. Mass calculated: 11767.6, mass found: 11767.3. This suggests that the conjugate was synthesized and purified correctly.

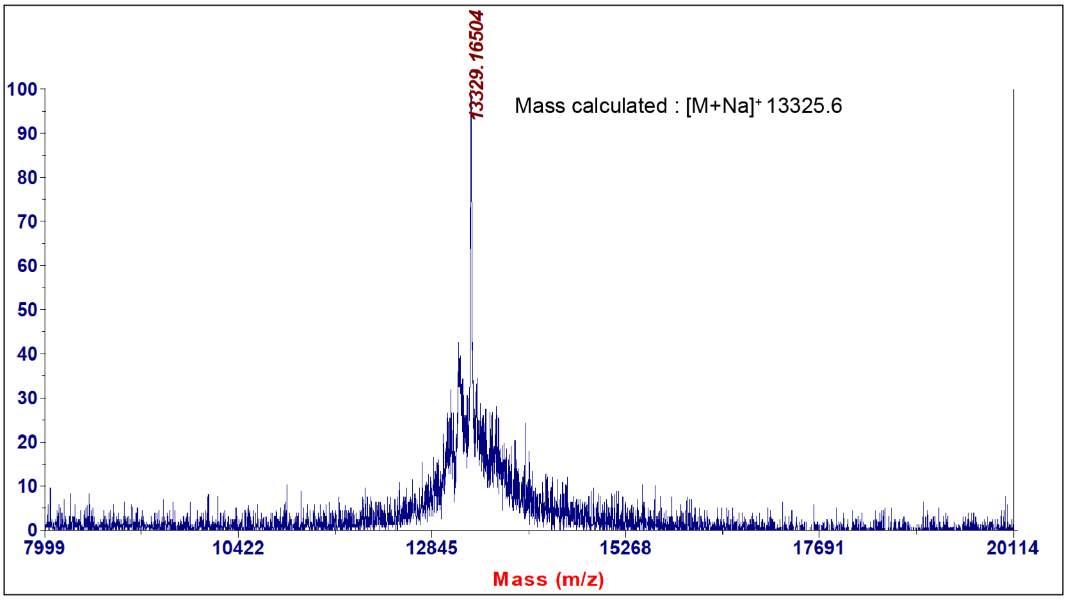

Figure S10. MALDI TOF mass spectrometry of L-Apt.4-1c-ASO15nt$_{(APP)}$ conjugate. Mass calculated:13325.6, mass found: 13329.2. This suggests that the conjugate was synthesized and purified correctly.

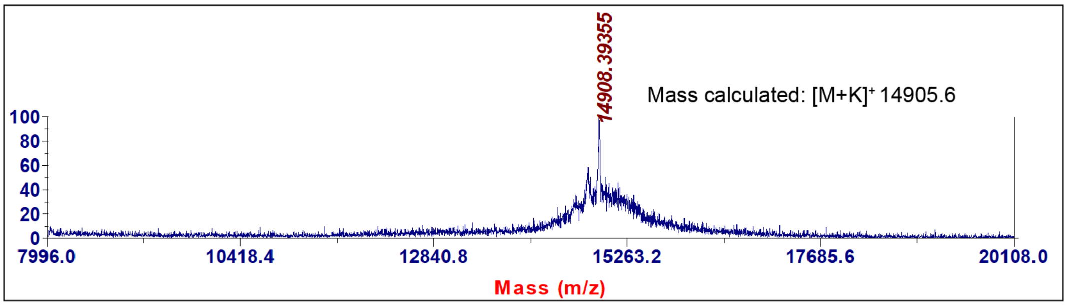

Figure S11. MALDI TOF mass spectrometry of L-Apt.4-1c-ASO20nt$_{(APP)}$ conjugate. Mass calculated: 14905.6, mass found: 14908.4. This suggests that the conjugate was synthesized and purified correctly.

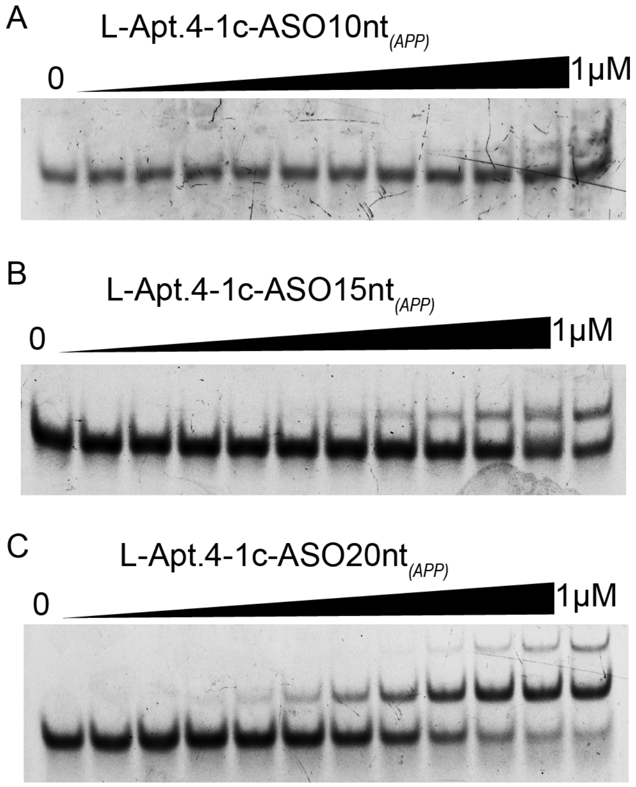

Figure S12. L-Apt.4-1c-ASO conjugates exhibit a markedly reduced binding capacity toward *APP* rG4 mut region analyzed by EMSA. (A-C) Binding of L-Apt.4-1c-ASO10nt*(APP)*, L-Apt.4-1c-ASO15nt*(APP)*, and L-Apt.4-1c-ASO20nt*(APP)* against HEX_*APP* rG4 mut region (10 nM) respectively analyzed by EMSA. The conjugates binding to *APP* rG4 mut region is much weaker than to *APP* rG4 wt region (compared to Fig. 2B-E), supporting the importance of the rG4 structure recognition mode. *APP* rG4 mut region used is 10 nM.

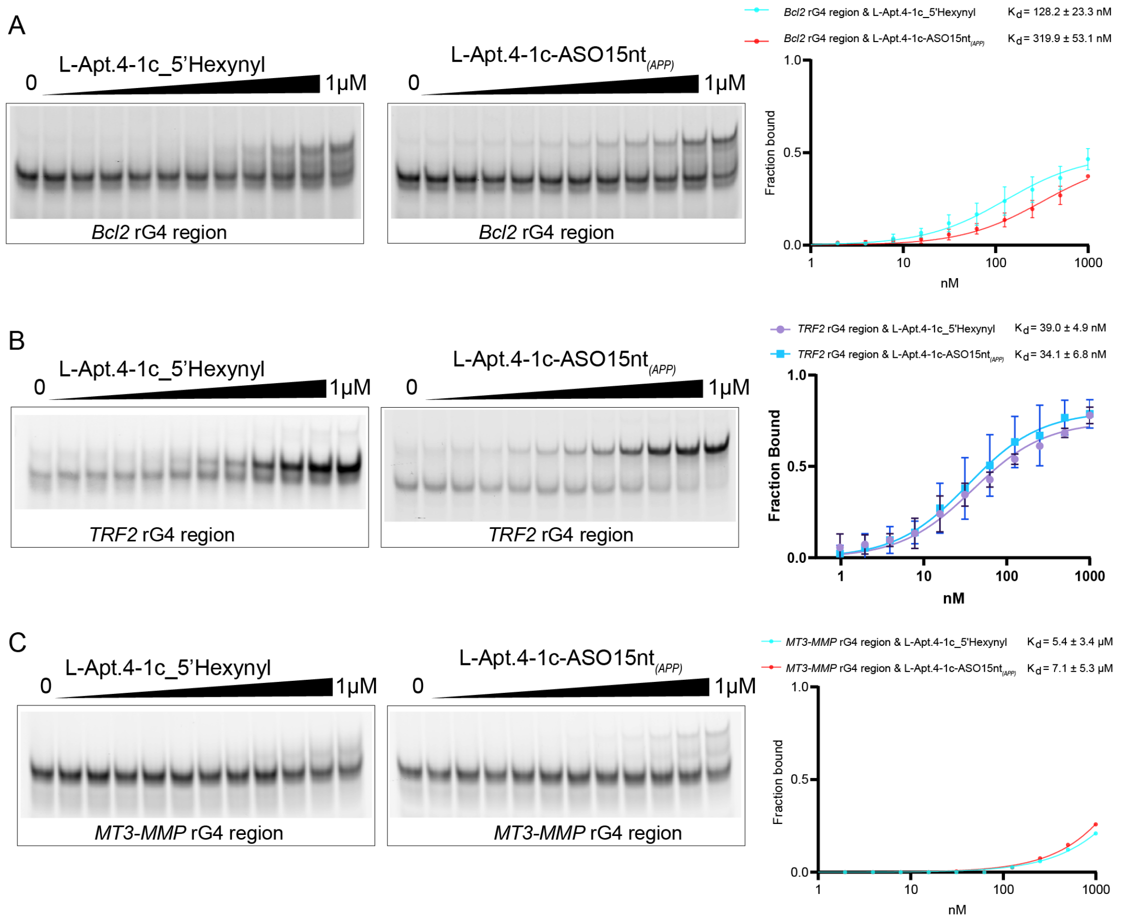

Figure S13. L-Apt.4-1c-ASO conjugate shows no enhanced binding to *Bcl2* rG4 region, *TRF2* rG4 region and *MT3-MMP* rG4 region analyzed by EMSA assay. (A) The K_ds for L-Apt.4-1c_5'Hexynyl and L-Apt.4-1c-ASO15nt*(APP)* against FAM_*Bcl2* rG4 region are determined to be 128.2 ± 23.3 nM and 319.9 ± 53.1 nM , respectively. (B) The K_ds for L-Apt.4-1c_5'Hexynyl and L-Apt.4-1c-ASO15nt*(APP)* against FAM_*TRF2* rG4 region are determined to be 39.0 ± 4.9 nM and 34.1 ± 6.8 nM, respectively. (C) The K_ds for L-Apt.4-1c_5'Hexynyl and L-Apt.4-1c-ASO15nt*(APP)* against FAM_*MT3-MMP* rG4 region are determined to be 5.4 ± 3.4 μM and 7.1 ± 5.3 μM, respectively. These results, together with Figure 2 and Figure S7, supported that L-aptamer-ASO strategy can improve the binding specificity and affinity to the intended rG4 target region, but not to the non-target rG4s. The oligos *of Bcl2* rG4 region, *TRF2* rG4 region and *MT3-MMP* rG4 region used are 10 nM.

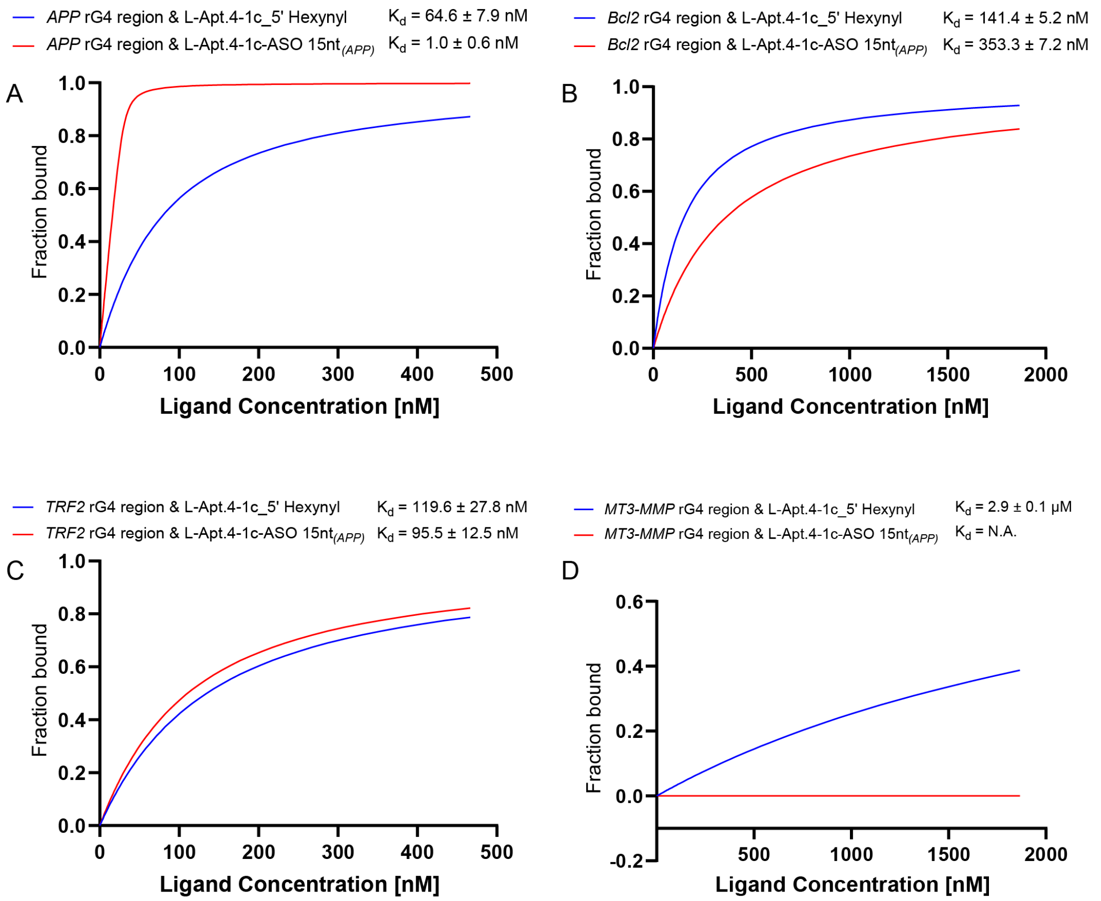

Figure S14. L-Apt.4-1c-ASO conjugate shows enhanced binding to *APP* D-rG4 region analyzed by MST assay. (A) The K_ds for L-Apt.4-1c_5'Hexynyl and L-Apt.4-1c-ASO15nt$_{(APP)}$ against FAM_*APP* rG4 wt region are determined to be 64.6 ± 7.9 nM and 1.0 ± 0.6 nM, respectively. (B) The K_ds for L-Apt.4-1c_5'Hexynyl and L-Apt.4-1c-ASO15nt$_{(APP)}$ against FAM_*Bcl2* rG4 region are determined to be 141.4 ± 5.2 nM and 353.3 ± 7.2 nM, respectively. (C) The K_ds for L-Apt.4-1c_5'Hexynyl and L-Apt.4-1c-ASO15nt$_{(APP)}$ against FAM_*TRF2* rG4 region are determined to be 119.6 ± 27.8 nM and 95.5 ± 12.5 nM, respectively. (D) The K_ds for L-Apt.4-1c_5'Hexynyl and L-Apt.4-1c-ASO15nt$_{(APP)}$ against FAM_*MT3-MMP* rG4 region are determined to be 2.9 ± 0.1 μM and N.A., respectively. N.A. means cannot be determined. These MST results are consistent with the EMSA results in Figure 2, Figure S7, and Figure S13, and support that L-aptamer-ASO strategy can improve the binding specificity and affinity to the intended rG4 target region, while not to the non-target rG4s. The oligos *of APP* rG4 region, *Bcl2* rG4 region, *TRF2* rG4 region and *MT3-MMP* rG4 region used are 30 nM.

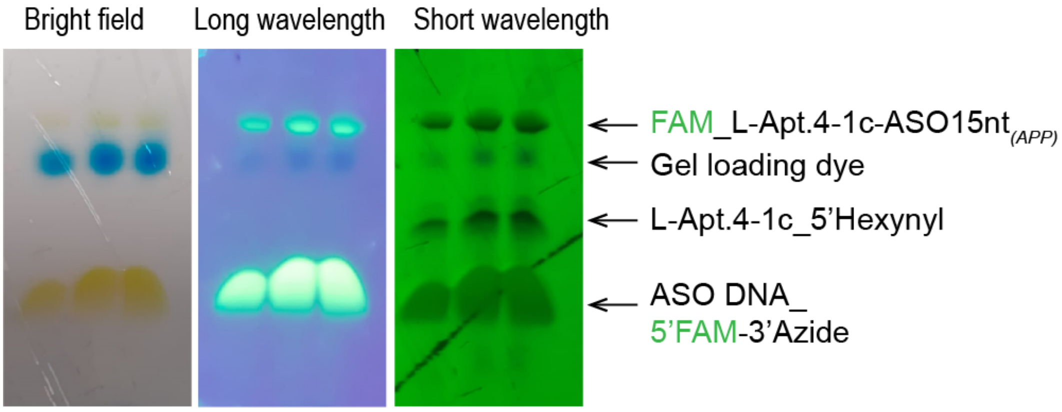

Figure S15. Synthesis of FAM_L-Apt.4-1c-ASO15nt*(APP)* conjugate for the specific recognition of *APP* rG4 region in cell imaging assay. Long wavelength is to show the FAM signal, while short wavelength is used to display all nucleic acid oligos. The FAM_L-Apt.4-1c-ASO15nt*(APP)* product was subsequently gel and column purified for in cell imaging assay (see methods).

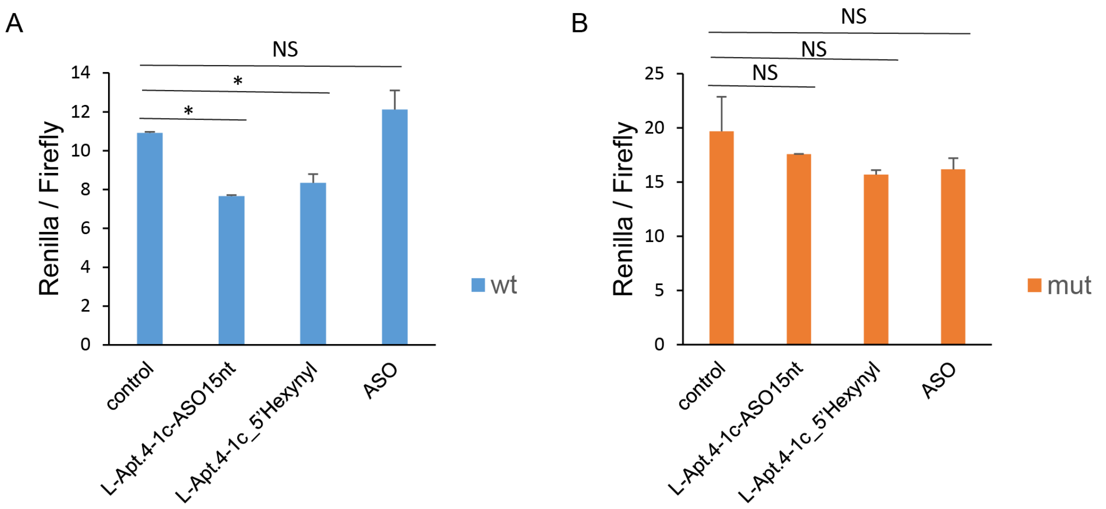

Figure S16. Dual luciferase reporter gene assay showed that L-Apt.4-1c-ASO15nt$_{(APP)}$ and L-Apt.4-1c_5'Hexynyl can negatively regulated gene expression via targeting *APP* rG4 structure. (A) Reporter gene assay result on wt construct, treated with / without L-Apt.4-1c-ASO15nt$_{(APP)}$, L-Apt.4-1c_5'Hexynyl or ASO, respectively. (B) Reporter gene assay result on mut construct, treated with / without L-Apt.4-1c-ASO15nt$_{(APP)}$, L-Apt.4-1c_5'Hexynyl or ASO, respectively. Error bars display standard error of mean. n=3; *, $P < 0.05$; NS, not significant.

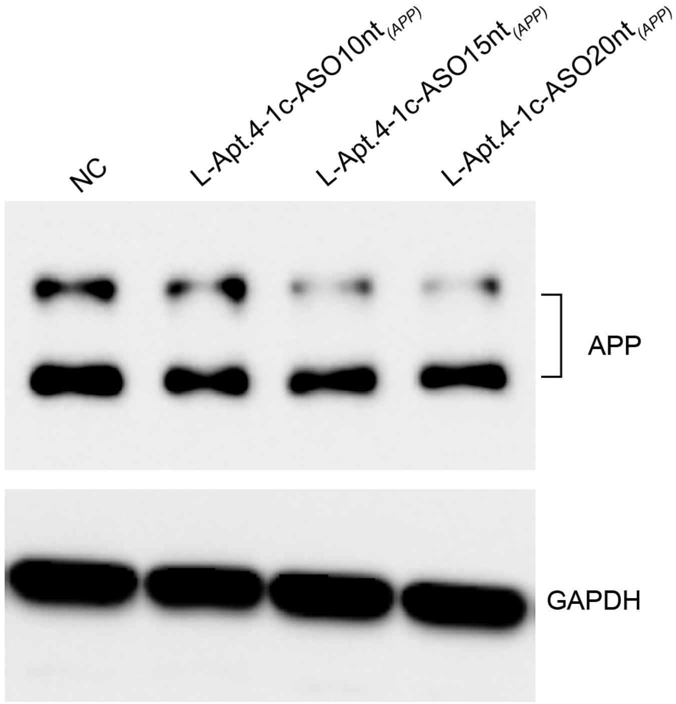

Figure S17. Inhibitory effect of L-Apt.4-1c-ASO10nt*(APP)*, L-Apt.4-1c-ASO15nt*(APP)*, and L-Apt.4-1c-ASO20nt*(APP)* on endogenous APP protein level in Hela cells detected by western blotting. NC, negative untreated control. L-Apt.4-1c-ASO15nt*(APP)* and L-Apt.4-1c-ASO20nt*(APP)* showed stronger inhibition effect on APP protein production. The concentration of conjugates is 30 nM. L-Apt.4-1c-ASO15nt*(APP)* was employed in subsequent cellular assays.

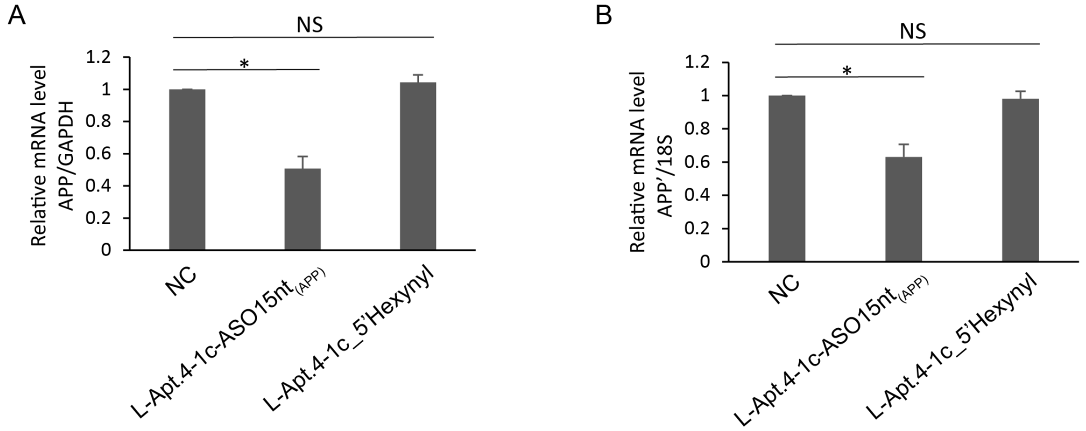

Figure S18. L-Apt.4-1c-ASO15nt*(APP)* conjugate knock down endogenous *APP* mRNA level in cells analyzed by RT-qPCR. GAPDH (A) or 18S rRNA (B) is used as loading control. The expression level of *APP* mRNA was significantly reduced in the L-Apt.4-1c-ASO15nt*(APP)*-treated group, whereas the level of *APP* mRNA in the L-Apt.4-1c_5'Hexynyl-treated group showed no significant difference in comparison with the control group (NC), suggesting that L-Apt.4-1c-ASO15nt*(APP)* exerts mRNA knockdown through an ASO-dependent manner by triggering RNase H cleavage in cells. Error bars display standard error of mean. n=3; *, $P < 0.05$; NS, not significant.

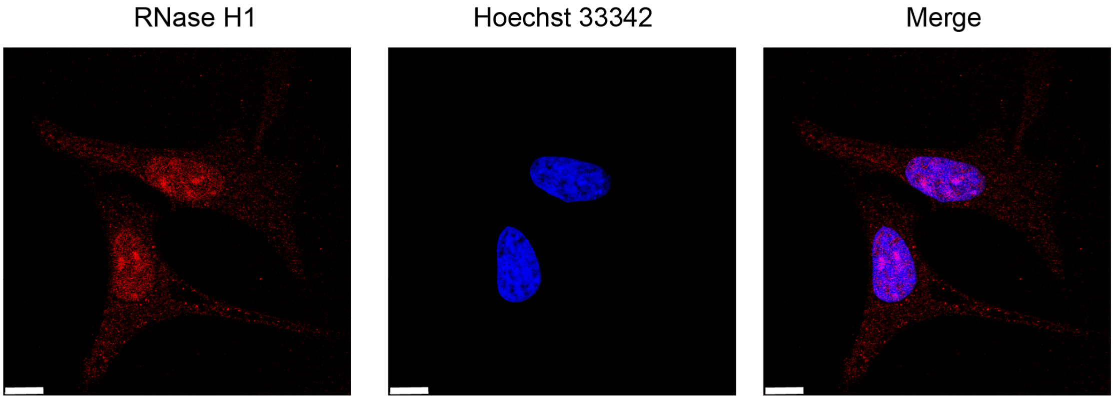

Figure S19. RNase H1 subcellular distribution analyzed by confocal microscopy. RNase H1 localized both in nucleus and cytoplasm. Hoechst 33342 is used to stain the nucleus in cells. Scale bar = 10 μm.

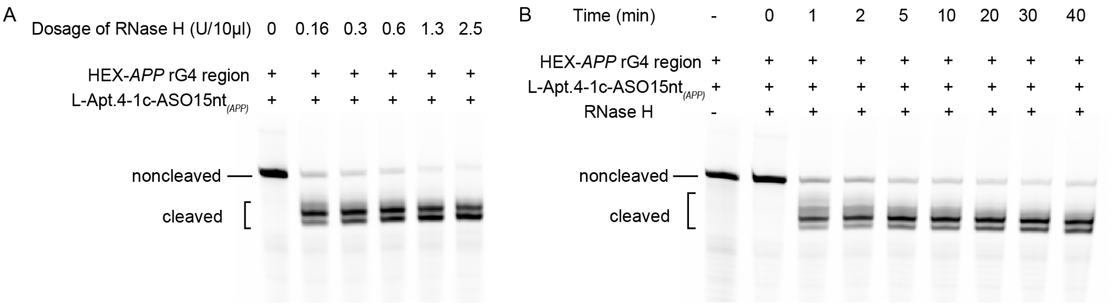

Figure S20. The cleavage of the conjugate-*APP* rG4 region complex by RNase H is contingent on the dosage levels and duration of exposure. (A) RNase H cleavage assay with different dosages of RNase H treatment detected by denaturing polyacrylamide gel. With RNase H dosage increasing, the intensity of the cleaved bands increases while that of noncleaved bands decrease, indicating that cleavage is dosage dependent. The reaction time is 20 min. (B) RNase H cleavage assay with different time of RNase H treatment detected by denaturing polyacrylamide gel. With RNase H treatment time increasing, the intensity of the cleaved bands increases while that of noncleaved bands decrease, indicating that cleavage is time dependent. The dosage of RNase H used is 0.3U / 10μl. HEX_*APP* rG4 region oligo used is 300 nM.

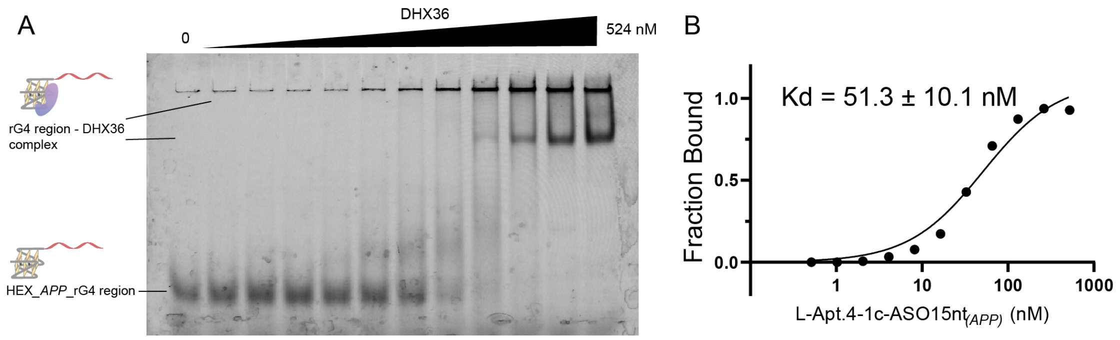

Figure S21. DHX36 protein binds well to *APP* rG4 region analyzed by EMSA. (A) With DHX36 concentration increasing, the intensity of the bound bands (rG4 region – DHX36 complex) increase while that of unbound bands (HEX_*APP* rG4 region) decrease, indicating that DHX36 interacts directly with *APP* rG4 region. (B) The K_d for DHX36 against *APP* rG4 region is determined to be 51.3 ± 10.1 nM. *APP* rG4 region used is 10 nM.

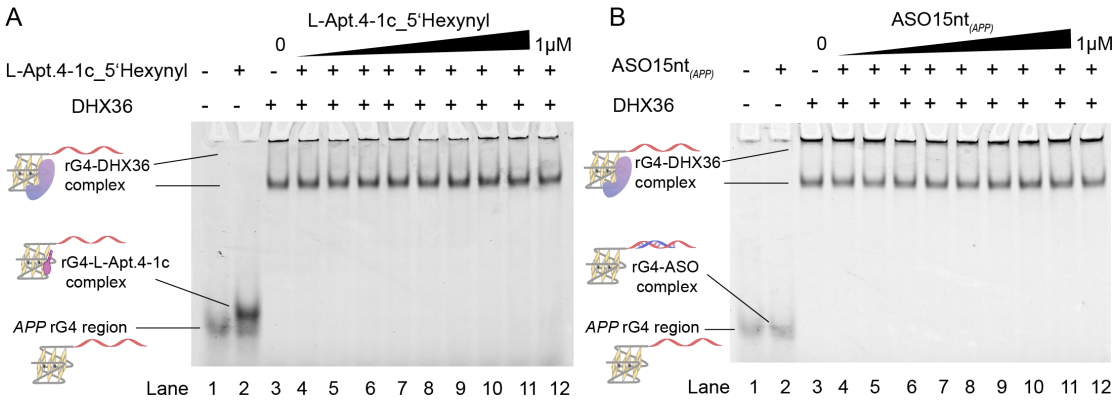

Figure S22. Inhibition assay shows that neither L-Apt.4-1c_5'Hexynyl nor ASO15nt*(APP)* can disrupt the interaction between *APP* rG4 region and DHX36 protein. HEX_*APP* rG4 region used is 5 nM.

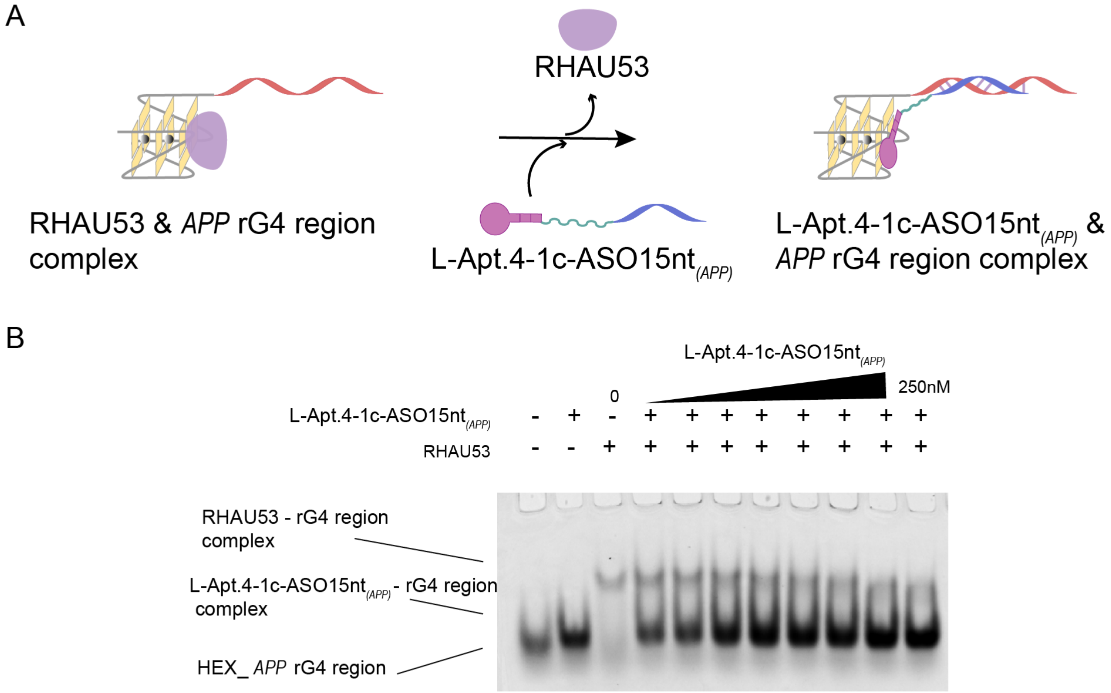

Figure S23. L-Apt.4-1c-ASO15nt*(APP)* conjugate can dissociate *APP* rG4 – RHAU53 interaction analyzed by EMSA. (A) Graphical representation of L-Apt.4-1c-ASO15nt*(APP)* conjugate to dissociate *APP* rG4 – RHAU53 peptide interaction. (B) Inhibition assay showed L-Apt.4-1c-ASO15nt*(APP)* conjugate can dissociate the interaction between HEX_*APP* rG4 region and RHAU53 peptide effectively. *APP* rG4 region used is 5 nM.

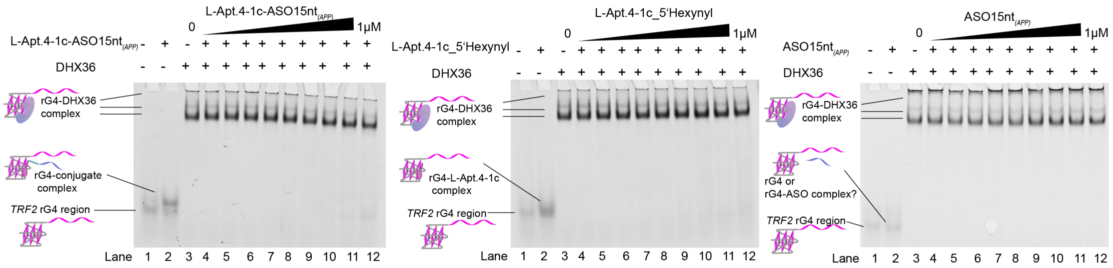

Figure S24. Inhibition assay shows that L-Apt.4-1c-ASO15nt$_{(APP)}$, L-Apt.4-1c_5'Hexynyl, and ASO15nt$_{(APP)}$ cannot disrupt the interaction between *TRF2* rG4 region and DHX36 protein. FAM_*TRF2* rG4 region used is 5 nM.

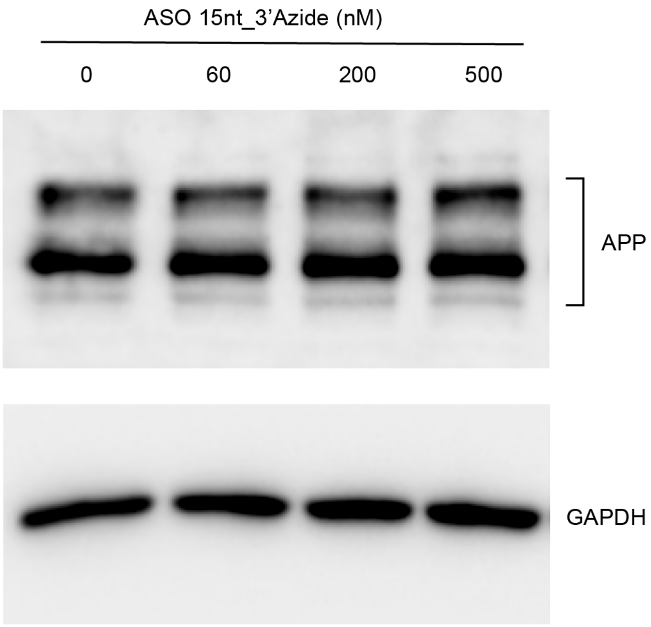

Figure S25. ASO itself cannot inhibit endogenous *APP* expression analyzed by western blotting. Hela cells were transfected with different concentration (0, 60, 200, and 500 nM) of ASO 15nt_3’Azide for 22 h.
